# VPS34 Governs Oocyte Developmental Competence by Regulating Mito/Autophagy: A Novel Insight into the Significance of RAB7 Activity and Its Subcellular Location

**DOI:** 10.1002/advs.202308823

**Published:** 2024-09-17

**Authors:** Wenwen Liu, Kehan Wang, Yuting Lin, Lu Wang, Xin Jin, Yuexin Qiu, Wenya Sun, Ling Zhang, Yan Sun, Xiaowei Dou, Shiming Luo, Youqiang Su, Qingyuan Sun, Wenpei Xiang, Feiyang Diao, Jing Li

**Affiliations:** ^1^ State Key Laboratory of Reproductive Medicine and Offspring Health Women's Hospital of Nanjing Medical University Nanjing Maternity and Child Health Care Hospital Nanjing Medical University Nanjing Jiangsu 211166 China; ^2^ State Key Laboratory of Reproductive Medicine and Offspring Health Center of Reproduction and Genetics Affiliated Suzhou Hospital of Nanjing Medical University Suzhou Municipal Hospital Gusu School Nanjing Medical University Suzhou Jiangsu 215002 China; ^3^ The Center for Clinical Reproductive Medicine State Key Laboratory of Reproductive Medicine and Offspring Health The First Affiliated Hospital of Nanjing Medical University Nanjing Jiangsu 212028 China; ^4^ State Key Laboratory of Reproductive Medicine and Offspring Health Nanjing Medical University Nanjing Jiangsu 211166 China; ^5^ Department of Reproductive Medicine Cangzhou Central Hospital Cangzhou Hebei 061012 China; ^6^ Department of Center of Reproductive Medicine Wuxi Maternity and Child Health Care Hospital Nanjing Medical University Wuxi Jiangsu 214200 China; ^7^ Institute of Reproductive Health Tongji Medical College Huazhong University of Science and Technology Wuhan Hubei 430074 China; ^8^ Department of Obstetrics and Gynecology The Second Affiliated Hospital of Nanjing Medical University Nanjing Medical University Nanjing Jiangsu 210011 China; ^9^ Guangzhou Key Laboratory of Metabolic Diseases and Reproductive Health Guangdong‐Hong Kong Metabolism & Reproduction Joint Laboratory Reproductive Medicine Center Guangdong Second Provincial General Hospital Guangzhou Guangdong 513023 China; ^10^ Shandong Provincial Key Laboratory of Animal Cells and Developmental Biology School of Life Sciences Shandong University Qingdao Shandong 266237 China; ^11^ Innovation Center of Suzhou Nanjing Medical University Suzhou 430074 China

**Keywords:** autophagy, mitophagy, oocyte, retromer, VPS34

## Abstract

Asynchronous nuclear and cytoplasmic maturation in human oocytes is believed to cause morphological anomalies after controlled ovarian hyperstimulation. Vacuolar protein sorting 34 (VPS34) is renowned for its pivotal role in regulating autophagy and endocytic trafficking. To investigate its impact on oocyte development, oocyte‐specific knockout mice (ZcKO) are generated, and these mice are completely found infertile, with embryonic development halted at 2‐ to 4‐cell stage. This infertility is related with a disruption on autophagic/mitophagic flux in ZcKO oocytes, leading to subsequent failure of zygotic genome activation (ZGA) in derived 2‐cell embryos. The findings further elucidated the regulation of VPS34 on the activity and subcellular translocation of RAS‐related GTP‐binding protein 7 (RAB7), which is critical not only for the maturation of late endosomes and lysosomes, but also for initiating mitophagy via retrograde trafficking. VPS34 binds directly with RAB7 and facilitates its activity conversion through TBC1 domain family member 5 (TBC1D5). Consistent with the cytoplasmic vacuolation observed in ZcKO oocytes, defects in multiple vesicle trafficking systems are also identified in vacuolated human oocytes. Furthermore, activating VPS34 with corynoxin B (CB) treatment improved oocyte quality in aged mice. Hence, VPS34 activation may represent a novel approach to enhance oocyte quality in human artificial reproduction.

## Graphical abstract

VPS34 plays a crucial role in maintaining mitophagy/autophagy flux during oocyte cytoplasmic maturation and early embryonic development. It exerts its regulatory function by modulating RAB7 activity and facilitating its mitochondrial translocation, thereby initiating mitophagy through interactions with the TBC1D5‐VPS26A‐retromer complex. The study indicates the application of a VPS34 activator holds promise as an innovative and effective approach to enhance oocyte quality during ovarian aging.

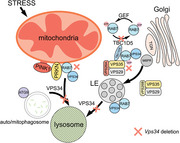

## 1 Introduction

The success of assisted reproduction technology (ART) treatment is dependent on oocyte quality. Unlike in vivo oocyte maturation, which occurs through a natural selection procedure, controlled ovarian hyperstimulation suppresses this procedure and allows oocytes that are destined to undergo atresia or regression to mature, albeit with inherently compromised quality.^[^ ^1^ ^]^ Oocyte maturation encompasses nuclear and cytoplasmic maturation, both of which are essential for the oocyte to acquire the capability for fertilization and early embryonic development. Nuclear maturity refers to the oocyte's capacity to resume and complete meiosis, while cytoplasmic changes, including organelle reorganization, mRNA transcription, protein translation, and post‐translational modification of proteins, collectively referred to as cytoplasmic maturation.^[^ ^2^, ^3^, ^4^ ^]^ Nuclear maturity alone is not enough to determine the competence of an oocyte. An oocyte with a deficiency in cytoplasmic maturity, on the other hand, could not be fertilized and drive subsequent embryo development. This could lead to fertilization failure, low‐pregnancy rate, abortion, or long‐term consequences in vivo.^[^ ^5^ ^]^

The maternal and paternal contributions to the embryo fate are not equal. Oocyte is the major determinant of embryo developmental competence. Since maturing oocyte are transcriptionally silent, the reorganization of organelle and cytoskeletal structure that are necessary for cytoplasmic maturation is directed by proteins and RNAs stored in the oocyte.^[^ ^3^ ^]^ During the transition from oocyte to embryo, the process which is referred as zygotic genome activation (ZGA), these maternal proteins and RNAs will be rapidly degraded and replaced finally by zygotic proteins and RNAs.^[^ ^6^ ^]^ Until now, the mechanisms that compromise oocyte developmental competence are still not clear. Autophagy is a cellular degradation and recycling process that is highly conserved from yeast to mammals.^[^ ^7^ ^]^ It has been reported germ cell‐specific deletion of *Atg7* (autophagy‐related protein 7) led to the subfertility in mice with severe ovarian follicle loss.^[^ ^8^ ^]^ Oocyte‐specific *Atg5* (autophagy‐related 5) knockout mice failed to develop beyond the four‐ and eight‐cell stages if they were fertilized by *Atg5*‐null sperm.^[^ ^9^ ^]^ The development of *Beclin1* null embryos was blocked after implantation at E7.5.^[^ ^10^ ^]^ Therefore, the significance of autophagy in oocyte and preimplantation embryonic development has recently been uncovered. Further investigation is necessary to elucidate the regulatory mechanisms governing autophagy during the process.

VPS34, also known as PIK3C3, is the sole member of Class III phosphatidylinositol 3‐kinase expressed in all examined eukaryotic organisms. By producing phosphatidylinositol‐3‐phosphate (PI3P), VPS34 functions not only in autophagosome formation and autophagy flux, but also plays pivotal roles in endosome formation and vesicle trafficking to the lysosome.^[^ ^11^ ^]^ Being present in different intracellular compartments, VPS34 acts as multiple roles through the activities of two complex. Complex I, composed of VPS34 (Vps34 in yeast), VPS15, BECN1 (Atg6 in yeast) and ATG14, is involved in the nucleation of the phagophore during autophagosome formation; complex II, in which the subunit ATG14 is replaced with UVRAG (Vps38 in yeast), is associated with endosome trafficking and functions on the fusion of autophagosome with lysosome.^[^ ^12^ ^]^ Rab5 and Rab7 GTPases belong to the largest family of small GTP‐binding proteins. Like other small GTPases, they switch between an inactive GDP‐bound state and an active GTP‐bound state. This switching is regulated by Rab Guanine Nucleotide Exchange Factors (GEFs) and Rab GTPase‐Activating Proteins (GAPs), which are essential for controlling the trafficking of cargo from early and late endosomes to the lysosome for degradation.^[^ ^13^, ^14^ ^]^ Both Rab5 and Rab7 activate the VPS34 PI3K to generate PI3P on endosomes. PI3P then binds to effectors containing PX (Phox homology) or FYVE domains, facilitating coordinated functions in various aspects of endosome maturation and trafficking.^[^ ^14^ ^]^ Meanwhile, to prevent the over activation of RAB5 and RAB7 GTPases, there is a negative feedback loop in which VPS34 inactivates these GTPases through recruitment of the TBC1D2 family of GAPs.^[^ ^15^, ^16^ ^]^ Other than endosome/lysosome trafficking, VPS34, along with RAB5 and RAB7 has also been reported to be necessary for retrograde trafficking of endocytic cargos from endosome to late Gogi. GTP‐bound RAB5 does not bind retromer directly but recruits VPS34 to catalyze the production of PI3P, which interacts with the PX domains of the sorting nexins.^[^ ^17^ ^]^ In contrast, RAB7 binds directly with VPS26A‐VPS35 retromer complex which the activity is regulated by a retromer‐associated RAB7‐specific GAP, TBC1D5.^[^ ^18^ ^]^ Interestingly, this control of RAB7 activity is not required for the recycling of retromer‐dependent cargoes. Nevertheless, it plays a crucial role for maintaining RAB7 mobility which is essential for the proper formation of autophagosome around damaged mitochondria during Parkin‐mediated mitophagy.^[^ ^19^ ^]^ Therefore, RAB7 activity and motility emerge as key factors linking membrane trafficking pathways with autophagy/mitophagy. However, it remains unclear whether VPS34 is involved in regulating this intricate process.

Constitutive endocytosis has been reported to occur in the mouse oocyte.^[^ ^20^, ^21^ ^]^ Inhibition of receptor‐mediated endocytosis completely blocked spontaneous meiotic consumption. Recent studies implicated the involvement of RAB family members in oocyte meiotic maturation through modulating actin network.^[^ ^22^ ^]^ For example, knockdown of *Rab5* in mouse oocytes resulted in abnormal spindle assembly and transition from prophase arrest to meiosis I.^[^ ^23^ ^]^ Deletion of *Rab7* in mouse oocytes, however, caused the failure of polar body extrusion and asymmetric division.^[^ ^24^ ^]^ Our recent study has unveiled the importance of maintaining active levels of RAB7 in oocyte meiosis by inhibiting the mitophagy‐related pathway.^[^ ^25^ ^]^ Thus, understanding how multiple vesicular trafficking events coordinate to acquire developmental competence during oocyte maturation is crucial.

Given its main roles in both autophagy and endosomal trafficking, VPS34 is important to maintain the homeostasis of different organs and cell types. As total deletion of *Vps34* in mice was lethal at early embryonic stage, in recent years, by using *Vps34* conditional knockout mouse models, the role of VPS34 in specialized mammalian cells and tissues are now being uncovered in muscle, heart, kidney, thyroid, platelets or Schwann cells.^[^ ^26^, ^27^, ^28^, ^29^, ^30^ ^]^ Our previous study also demonstrated the important role of VPS34 in maintaining the polarity of Sertoli cells, which is essential for male fertility.^[^ ^31^ ^]^ In the study, we generated *Vps34* oocyte‐specific knockout mice by crossing with *Zp3*‐Cre mice to explore its regulatory mechanisms in growing oocyte. Our results revealed the important role of VPS34 in regulating oocyte cytoplasmic maturation. Mechanistically, it not only regulates RAB7 activity, but also participates in RAB7‐mediated recruitment of retromer on late endosomal and RAB7's shift during mitophagy initiation, which is an innovative finding. The deficiency of auto/mitophagy leads to the dysregulation of maternal protein degradation and finally the embryonic development arrest after oocyte fertilization.

## 2 Results

### 2.1 Deletion of Vps34 in Growing Oocytes Resulted in Infertility Due to Defects on Early Embryonic Development

**Figure 1:**
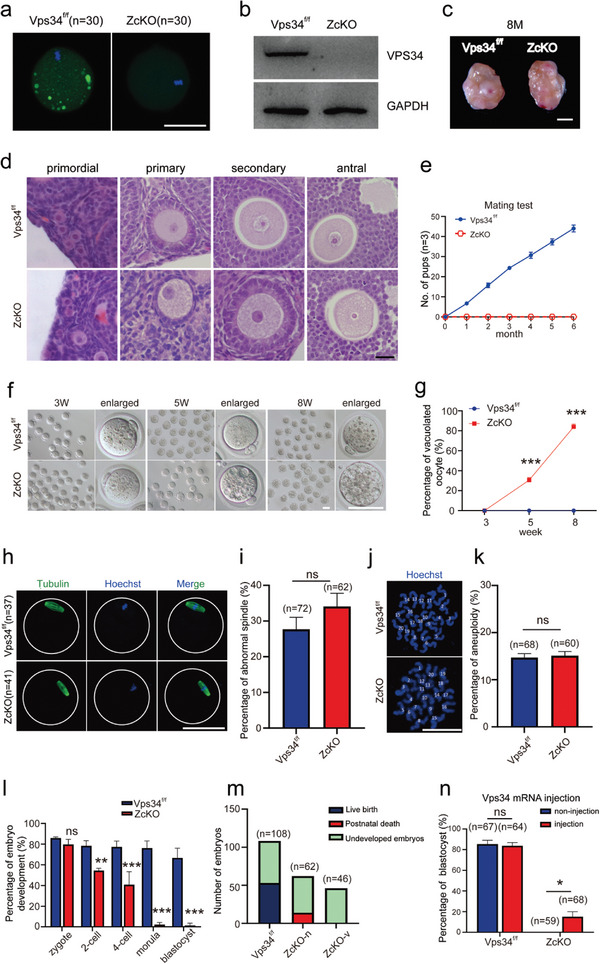
Deletion of VPS34 in growing oocytes caused infertility due to defects on early embryonic development. a) Immunofluorescence of VPS34 in MII oocytes collected from 3 W control (Vps34^f/f^) and ZcKO mice. Scale bar = 50 µm. b) Western blot analysis of the expression of VPS34 in control and ZcKO MII oocytes. The expression of GAPDH was served as the internal control. c) Ovarian morphology of 8 M ZcKO mice compared with littermate control mice. Scale bar = 1 mm. d) Morphology of ovarian follicles at different developmental stages from 5 W control and ZcKO mice. Scale bar = 10 µm. e) Fertility curves of control and ZcKO mice after 6 months of mating. f,g) Increased vacuolation observed in ZcKO oocytes collected at 3, 5, and 8 W of age. Representative MII oocytes at various ages are presented (f), along with the percentage of vacuolated oocytes in each group (g). Scale bar = 50 µm. h) Immunofluorescence of Tubulin to label oocyte spindle in MII oocytes collected from control and ZcKO mice at 5 W of age. Green, Tubulin; blue, Hoechst 33342. Scale bar = 50 µm. i) Percentage of oocytes with abnormal spindles in control and ZcKO oocytes. j) Staining of the spread chromosome with Hoechst 33342 in mature oocytes. Scale bar = 50 µm. k) Aneuploidy assessment after chromosome counting. l) Percentage of early embryonic development in the two groups. Oocytes were collected from 5Wcontrol and ZcKO mice for IVF and early embryonic development was followed. m) Live birth after embryo transfer. The 2‐cell embryos derived from ZcKO oocytes were classified as vacuolated (ZcKO‐v) and non‐vacuolated (ZcKO‐n) embryos before being transferred into foster mother mice for development until birth. Embryos that did not result in live births were considered undeveloped, while pups that died immediately after birth were classified as postnatal deaths. n) The assessment of embryonic development after injection of *Vps34* mRNA into control and ZcKO zygotes. At least 3 independent replicates were performed for each experiment. All graphs are presented as the means ± SEM. *p < 0.05, **p < 0.01, ***p < 0.001. ns = no significance.

To investigate the role of VPS34 during oocyte development, we generated a mouse line with conditionally deleted *Vps34* in growing oocytes by crossing female *Vps34^flox/flox^* (Vps34^f/f^) mice with *Zp3*‐Cre male transgenic mice, which we referred as *Vps34* ZcKO mice.^[^ ^32^ ^]^ Both immunofluorescence and Western blot analyses revealed that VPS34 protein was virtually absent in MII oocytes of ZcKO mice (**Figure** **1**a,b). We then followed ovarian development until 8 M of age (Figure 1c) and no difference on ovarian weight and follicle distributions (Figure S1a, Supporting Information) were observed in ZcKO mice except that we found growing oocytes were full of vacuoles since 5 W of age (Figure 1d). Next, the fertility test was performed by crossing Vps34^f/f^ (control) or ZcKO female mice (8 W of age) with fertile males, however, the ZcKO mice were completely infertile with no offspring produced after 6 months of fertility test (Figure 1e). We obtained mature oocytes from 3, 5 and 8 weeks of control and ZcKO mice through superovulation. Although no difference on ovulated oocyte numbers between the two groups (Figure S1b, Supporting Information), vacuolated oocytes were found started from 5 W‐ZcKO mice (30.67%) and the proportion increased to 84.01% in 8 W‐ZcKO oocytes (Figure 1f,g). When oocytes from 5 W of control and ZcKO mice were for tubulin staining and chromosome expansion test, however, no significant difference on spindle morphology and aneuploidy was observed between the two groups (Figure 1h–k). The results indicate *Vps34* deletion in oocyte‐induced cytoplasmic vacuolization but has no effect on oocyte meiosis or nuclear maturation. We then collected zygotes from 5 W control and ZcKO mice and cultured in vitro for 96 h to follow early embryonic development. The results demonstrated decreased development of *Vps34*‐deleted oocytes to 2‐cell stage and most of embryos were arrested at the 4‐ to 8‐cell stage (Figure 1l). To see if the vacuolization of oocyte affects the embryonic developmental potency, we divided vacuolated and non‐vacuolated oocytes from 5‐week‐old ZcKO mice for in vitro fertilization and transferred derived 2‐cell embryos into foster mice. The results showed that non‐vacuolated oocytes could be fertilized and produced offspring after embryonic transfer, but the chance is very low and the offspring died shortly after birth, while vacuolated oocytes had no offspring delivered at all (Figure 1m). When we attempted to rescue the disorders of embryonic development in ZcKO mice by microinjecting fertilized zygote with *Vps34* mRNA (Figure S1c, Supporting Information), we found although some of embryos could develop to blastocyst, the percentage (15%) was significantly lower compared to over 80% blastocyst development in control wild type mice (Figure 1n). These results suggest the maternal effect of VPS34 on early embryonic development, in which the deficiency in oocytes cannot be remedied only in zygotes.

### 2.2 Transcriptome Sequencing Revealed Differential Gene Expressions in Vps34 Deleted Oocytes and Derived Embryos

**Figure 2:**
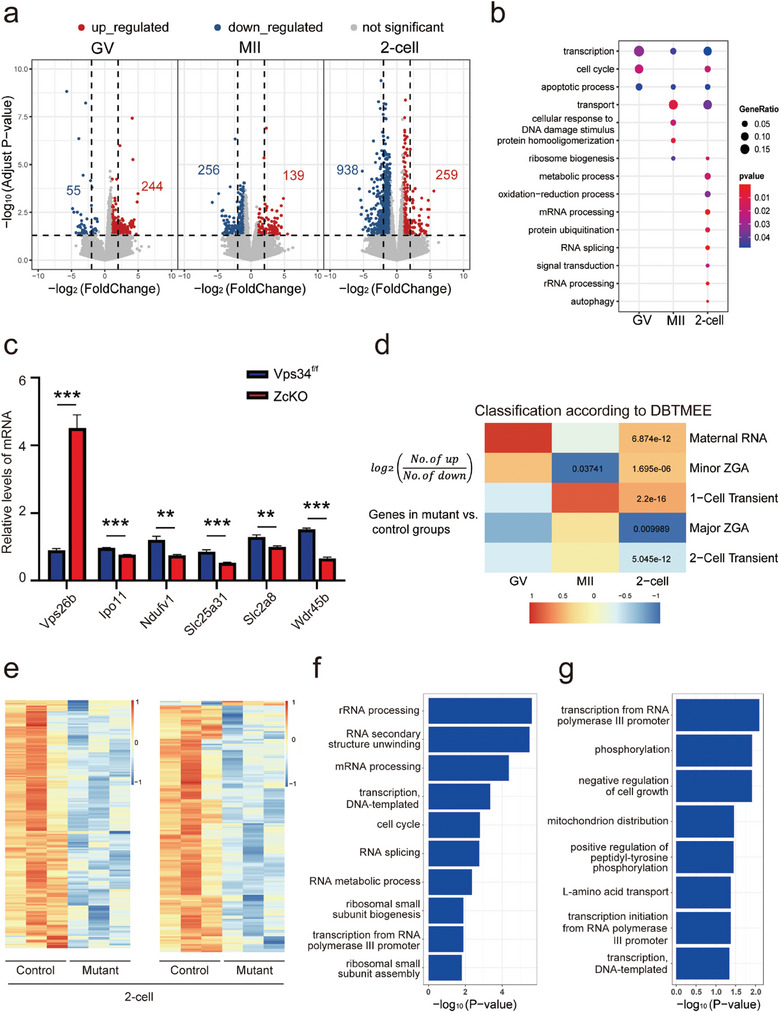
Transcriptomic alterations in ZcKO oocytes and embryos. GV, MII, and 2‐cell embryos were collected from control and ZcKO mice at 5 W of age for single‐cell sequencing. a) Volcano plots of DEGs comparing the control and ZcKO groups. A number of upregulated and downregulated genes are represented separately in red and blue, respectively, with an absolute fold change > 2 and *P*‐value < 0.05. b) GO enrichment analysis of DEGs in GV, MII oocytes, and 2‐cell embryos. The size and color of dots represent the ratio of genes in the term and *P*‐value separately. c) Validation of DEGs enriched in transport and autophagy pathways by RT‐PCR on MII oocytes collected from control and ZcKO mice. At least 3 independent replicates were performed for each experiment. All graphs are presented as the means ± SEM. *p < 0.05, **p < 0.01, ***p < 0.001.d) Comparison of DEGs against a public database (DBTMEE) in which genes are categorized by specific expression patterns at any developmental stage. The Heatmap shows the log_2_ ratio of the number of upregulated genes to downregulated genes in the ZcKO group compared to the control group at GV, MII oocyte and 2‐cell embryo stages. Fisher's exact test performed with statistically significant *P*‐values labeled. e) The Heatmap of DEGs derived from 2‐cell embryos, showing overlap with the major ZGA cluster (left) and the 2‐cell transient activation cluster (right) in the DBTMEE database. f,g) GO enrichment analysis of DEGs overlapped in the major ZGA cluster (f) and the 2‐cell transient activation cluster (g). The size and color of dot represent the number of genes in the term and ‐log_10_ (*P*‐value) separately.

To investigate the transcriptional alterations caused by the absence of *Vps34*, we conducted transcriptome profiling of GV and MII oocytes, as well as 2‐cell embryos from control and ZcKO mice. Compared with a control group, a total of 299 and 395 differential expressed genes (DEGs) were enriched in GV and MII oocytes of ZcKO mice, respectively. In 2‐cell embryos derived from ZcKO oocytes, however, large amounts of genes (1197) were differentially expressed, of which 259 genes were up‐regulated and 938 genes were down‐regulated (**Figure** **2**a). Gene ontology (GO) analysis of DEGs in GV, MII oocytes, and 2‐cell embryos was shown in Figure 2b, in which transcription, apoptosis, transport or autophagy‐related genes were commonly enriched in *Vps34* deleted oocytes or embryos. Some DEGs related with transport or autophagy were chosen for RT‐PCR in control and ZcKO MII oocytes in which we found the extremely increased expression of *Vps26b*, a distinct component of retromer complex and a significant decrease of *Wdr45b*, a member belong to a WD40 repeat‐containing PI3P‐binding protein family in ZcKO oocytes (Figure 2c). *Wdr45* and *Wdr45b* are homologs of yeast *Atg18* and are critical for autophagosome‐lysosome fusion in neural cells.^[^ ^33^ ^]^ Thus, the results suggest the defects of endosome or autophagy‐related functions in *Vps34* deleted oocytes and derived embryos.

In mice, zygotic genomic activation (ZGA), including minor ZGA or major ZGA, are required for embryonic development beyond the 2‐cell stage.^[^ ^34^ ^]^ Since most embryos derived from *Vps34* deleted oocytes are hardly developed beyond 4‐cell stage, we compared the DEGs of 2‐cell sequencing data against a public database of early mouse embryonic transcriptomes (a database of transcriptome in mouse early embryos, DBTMEE),^[^ ^35^ ^]^ where genes were categorized according to their specific expression patterns.^[^ ^36^ ^]^ We observed the enrichment of maternal transcript clusters (maternal RNA and minor ZGA) and the defect of zygotic transcript clusters (major ZGA and two‐cell transient activation) in mutant 2‐cell embryos (Figure 2d). Heatmaps showed overlapped DEGs in the major ZGA cluster and in the 2‐cell transient activation cluster (Figure 2e), indicating that these genes are largerly downregulated in mutant 2‐cell embryos. GO analysis of these downregulated genes revealed their participation in ribosome biogenesis, mRNA processing, transcription and so on (Figure 2f,g). Subsequent RT‐PCR results confirmed significantly decreased expression of *Ppp1ca*, *Gabra1* (associated with ZGA), *Nsun5* and *Gprasp2* (related to the 2‐cell stage) in mutant 2‐cell embryos. Notably, a partial rescue of gene expression was observed following microinjection of *Vps34* mRNA into ZcKO zygotes (Figure S1d, Supporting Information). Additionally, 2‐cell embryos collected from control and ZcKO mice were stained with annexin‐V or labeled with EU and EdU to evaluate the difference on cellular apoptosis, RNA and DNA synthesis. Both apoptotic signals (Figure S2a,b, Supporting Information) and EdU labeling (Figure S2e,f, Supporting Information) showed no differences between the two groups, however, the intensified EU labeling (Figure S2c,d, Supporting Information) was observed in ZcKO embryos, which means abnormal transcriptional activity induced by maternal deficiency of *Vps34*. On the contrary, the global protein synthesis assay demonstrated a significant decrease on protein synthesis in ZcKO mice (Figure S2g,h, Supporting Information). Due to the central role of mTOR signaling on autophagy and its interactions with VPS34, we examined the PI3K/mTOR signaling pathway in MII oocytes and 2‐cell embryos from control and ZcKO mice (Figure S2i,j, Supporting Information). Except for the significant decrease of p‐AKT levels in both ZcKO oocytes and 2‐cell embryos, our results revealed the activation of mTOR signaling pathway especially in ZcKO 2‐cell embryos which was shown by significantly increased phosphorylation of P70 and RPS6. The results indicate the disturbed protein synthesis in 2‐cell embryos after deleting *Vps34* in oocytes. Taken together, RNA sequencing analysis suggests that embryos derived from *Vps34* deleted oocytes suffered serious impairment on maternal‐zygotic transition (MZT), as reflected by the abnormal accumulation of maternal transcripts, the failure to activate zygotic transcripts, and the aberrant protein synthesis.

### 2.3 Vps34 Deficiency Led to the Blockage of the Autophagy Flux in Oocytes

**Figure 3:**
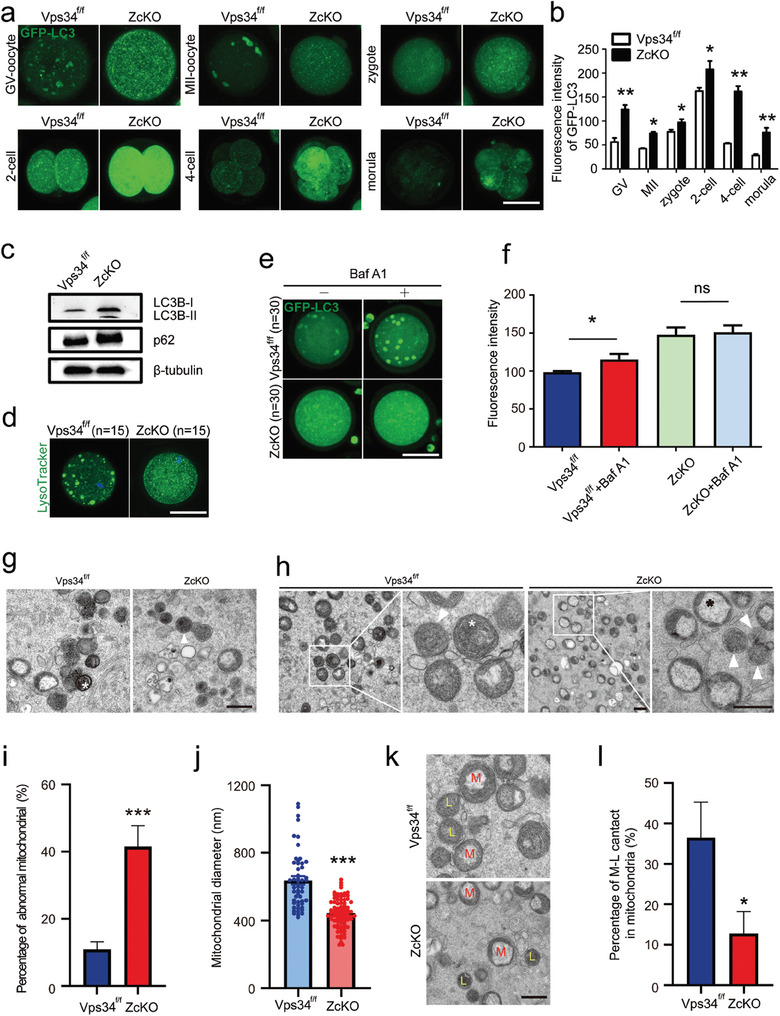
Blockage of autophagic flux in ZcKO oocytes and early embryos. a) Immunofluorescence of GFP‐LC3 in oocytes and early embryos obtained from GFP‐LC3‐Vps34^f/f^ (Vps34^f/f^) and GFP‐LC3‐ZcKO (ZcKO) mice. Oocytes were collected from GFP‐LC3‐Vps34^f/f^ (Vps34^f/f^) and GFP‐LC3‐ZcKO (ZcKO) mice at 5 W of age for IVF. Early embryos at different developmental stages were then collected to observe autophagic flux and autophagy punctae (autophagosomes). Scale bar = 50 µm. b) Intensity of GFP‐LC3 fluorescence compared between GFP‐LC3‐Vps34^f/f^ (Vps34^f/f^) and GFP‐LC3‐ZcKO (ZcKO) groups. c) Western blot of LC3B and p62 in control and ZcKO oocytes. β‐tubulin was used as an internal control. d) LysoTracker staining of oocytes collected from control and ZcKO mice. Scale bar = 50 µm. e) Fluorescence of LC3 GFP in GFP‐LC3‐Vps34^f/f^ (Vps34^f/f^) and GFP‐LC3‐ZcKO (ZcKO) oocytes after treatment with autophagy inhibitor Baf‐A1 for 4 h. Scale bar = 50 µm. f) Fluorescent densities before/after Baf‐A1 treatment in each group of oocytes. g) Different lysosomal status in control and ZcKO oocytes observed by TEM. The white asterisk represents the formation of autolysosome with organelles in control oocytes, while white triangle indicates an unfused lysosome. Scale bar = 500 nm. h) Mitochondrial morphology in control and ZcKO oocytes under TEM. The right panel of each group displays a magnified view of the white frame shown in the left panel. White triangles, unfused lysosomes; white asterisk, normal mitochondria; black asterisk, damaged mitochondria. Scale bar on the left = 500 nm. Scale bar on the right = 200 nm. i) Percentage of abnormal mitochondria in control and ZcKO oocytes. j) Measurement of mitochondrial diameter in control and ZcKO oocytes by ImageJ software. k) Representative distributions of lysosomes and mitochondria in control and ZcKO oocytes. M, mitochondria; L, lysosomes. Scale bar = 500 nm. l) Quantification of mitochondria closely interacting with lysosomes in control and ZcKO oocytes. *p < 0.05, **p < 0.01, ***p < 0.001. ns = no significance.

One of the reported functions of VPS34 in intracellular vesicle transport is the induction of autophagy on nutrient deprivation.^[^ ^37^ ^]^ Since autophagy related DEGs were enriched in *Vps34* deleted oocytes and derived embryos, we constructed GFP‐LC3 transgenic ZcKO mice to monitor autophagy status in oocytes and during early embryonic development. As shown in **Figure** **3**a, the LC3 GFP signal was detected as aggregated puncta (autophagosome) in GV and MII oocytes from GFP‐LC3‐Vps34^f/f^ (Vps34^f/f^) and after fertilization, the signal diffused in cytoplasm as mini dots with fluctuate fluorescence intensity during early embryonic development. However, such aggregates in oocytes were disappeared in GFP‐LC3‐ZcKO (ZcKO) mice and the fluorescence intensity was kept in a relative high level until 4‐cell to morula stage as compared with control oocytes and embryos (Figure 3b). Western blots showed the increased expression of autophagy markers LC3B I/II and p62 in *Vps34* deleted oocytes (Figure 3c). To see if the autophagy flux was affected in ZcKO oocytes, we labeled lysosomes by LysoTracker and similar as the distribution of LC3 GFP signals, the aggregated lysosomes was observed in control oocytes, while in ZcKO oocytes, the signals diffused in cytoplasm (Figure 3d). We then collected GV oocytes from GFP‐LC3‐Vps34^f/f^ (Vps34^f/f^) and GFP‐LC3‐ZcKO (ZcKO) mice and incubated them with 10 nM lysosomal inhibitor bafilomycin A1 (Baf‐A1) for 4 h to block autophagy flux. Figure 3e,f showed the increase of autophagosomes in GFP‐LC3‐Vps34^f/f^ (Vps34^f/f^) oocytes but still no aggregated LC3 GFP signals were observed in GFP‐LC3‐ZcKO (ZcKO) oocytes. The results indicate the defect on autophagosome formation and the blockage of autophagy flux in ZcKO oocytes. Under transmission electron microscopy (TEM), we could see the aggregates of mature lysosomes in control oocytes as shown by the engulfed organelles in them, however, only primary lysosomes were detected in ZcKO oocytes (Figure 3g). Meanwhile, we observed large amounts of impaired mitochondria scattered in ZcKO oocytes as shown by lack of electron density, cristae disappearance and vacuolization (Figure 3h,i). As compared with mitochondria in control oocytes, these damaged mitochondria were much smaller (Figure 3j) and we could observe primary lysosomes surrounded them (Figure 3k). However, fewer contacts were found between mitochondria and lysosomes (Figure 3l). These results suggest the deletion of *Vps34* induced the aberrant lysosome function which failed to fuse with autophagosome or engulf damaged mitochondria for degradation.

### 2.4 Vps34 Deficiency‐Induced Mitochondria Dysfunction and the Failure to Initiate Mitophagy

**Figure 4:**
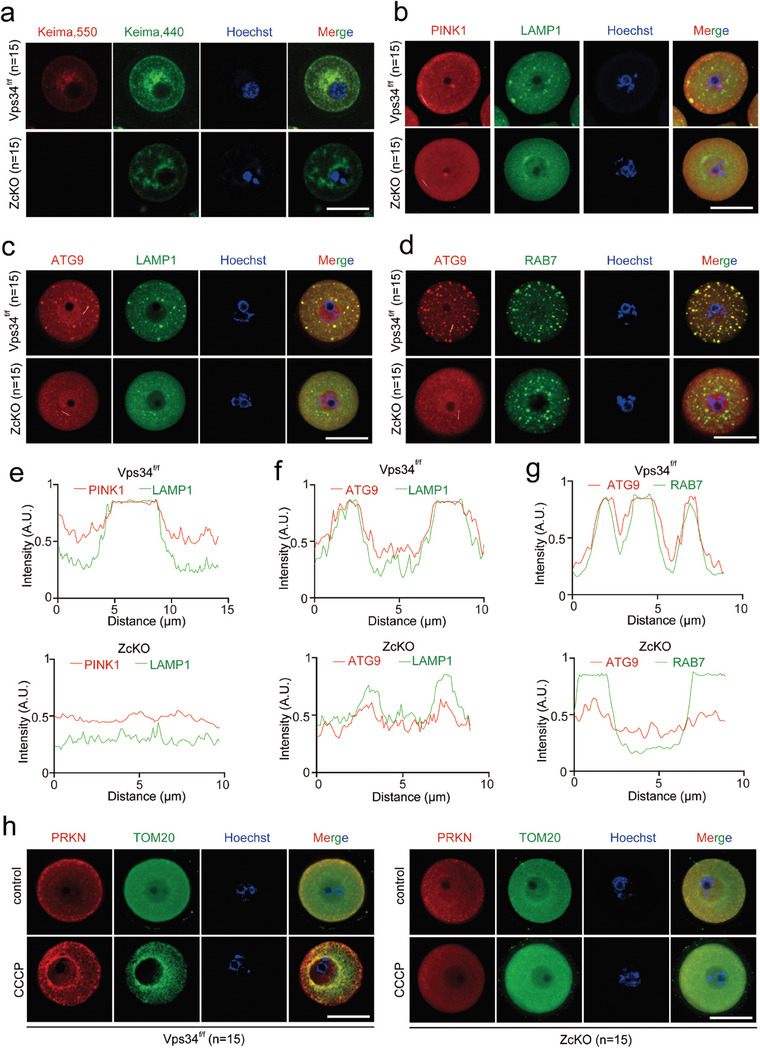
Blockage of mitophagy flux in ZcKO Oocytes. GV oocytes were collected from 5 W control and ZcKO mice after PMSG treatment for 48 h. a) Mitophagy flux with mt‐Keima labeling. GV oocytes from control and ZcKO mice were micro injected with mt‐Keima mRNAs and hold in 2 µM milrinone for 8–10 h. Images of oocytes were collected at fluorescent emission 550 nm (red) and 440 nm (green), respectively. Red indicates active mitophagic flux, representing the fusion of mitophagosome with lysosome. Scale bar = 50 µm. b) Immunofluorescent co‐staining of PINK1 (red) and LAMP1 (green) in control and ZcKO oocytes. Scale bar = 50 µm. c) Immunofluorescent co‐staining of ATG9 (red) and LAMP1 (green) in control and ZcKO oocytes. Scale bar = 50 µm. d) Immunofluorescent co‐staining of ATG9 (red) and RAB7 (green) in control and ZcKO oocytes. Scale bar = 50 µm. e‐g) Intensity measurement of the lines shown inside the oocytes in Figure b‐d. h) Immunofluorescent co‐staining of PRKN (red) and TOM20 (green) in control and ZcKO oocytes. Oocytes were treated with or without 100 µM CCCP for 1 h. Scale bar = 50 µm. Blue, nuclear staining with Hoechst 33342; Merge, representing a superposition of fluorescence signals from three channels.

Since massive mitochondria damage being observed in ZcKO oocytes by TEM, we then evaluated mitochondria functions in GV (Figure S3a, Supporting Information) and MII (Figure S3b, Supporting Information) oocytes by MitoTracker and the abnormal distribution of mitochondria was found in nearly all the checked ZcKO oocytes no matter with or without vacuoles (Figure S3c,d, Supporting Information). In addition, compared with control oocytes, ZcKO mouse oocytes had higher reactive oxygen species (ROS) levels (Figure S3e,f, Supporting Information) and significantly decreased mitochondrial membrane potential as shown by JC‐1 staining (Figure S3g,h, Supporting Information). Although there was no obvious change of ATP levels in GV oocytes from ZcKO mice, the ATP levels significantly decreased in ZcKO MII oocytes and derived 2‐cell embryos (Figure S3i, Supporting Information). Our previous study has revealed the importance of active mitophagy in GV oocytes in maintaining mitochondria quality for oocyte maturation and oocyte developmental competence.^[^ ^25^ ^]^ In the following experiments, we microinjected mt‐Keima into control and ZcKO GV oocytes, from the colorful change of Keima in mitochondria (green) or lysosome (red), the active mitophagy flux could be observed in control oocytes, however, the process of mitophagy was completely blocked in ZcKO oocytes (**Figure** **4**a; Figure S3j, Supporting Information). Western blots were then performed to check the expressions of PINK1 and PRKN, the key molecules of the mitophagy pathway.^[^ ^38^ ^]^ The results showed that the expression of PINK1 protein in ZcKO oocytes was significantly increased, while PRKN remained unchanged (Figure S3k, Supporting Information). Immunofluorescence of PINK1 with LAMP1 demonstrated the colocalization of PINK1 with LAMP1 to form mature lysosome in control oocytes, but in ZcKO oocytes, all the signals diffused in the cytoplasm, indicating the failure to transport damaged mitochondria to lysosome (Figure 4b,e). The defects on the autophagosome/mitophagosome formation were also manifested by the disappearance of ATG9/LAMP1 double‐positive puncta in the cytoplasm of ZcKO oocytes (Figure 4c,f). ATG9 is the only known membrane‐spanning autophagy protein which has been reported to play an important role for mitophagosome formation.^[^ ^39^ ^]^ Since RAB7, a small GTPase that controls transport to late endosomes and lysosomes, also functions in mitophagosome formation by targeting ATG9 vesicles,^[^ ^40^, ^41^ ^]^ we performed co‐staining of ATG9 and RAB7 in GV oocytes and similarly, we found the loss of ATG9 vesicles in oocytes but only RAB7 positive spots existed in the cytoplasm of ZcKO oocytes (Figure 4d,g). In addition, we observed partly overlapped staining of VPS34 and TOM20 (Translocase of Outer Mitochondria Membrane 20) with RAB7 in control oocytes, but no such phenomenon was found in ZcKO oocytes (Figure S4a–c, Supporting Information). These results suggest *Vps34* deletion induces RAB7 dislocation during autophagosome/mitophagosome formation. GV oocytes of control and ZcKO mice were also incubated with CCCP to induce mitophagy and then double stained with PRKN and TOM20 to label damaged mitochondria. As shown in Figure 4h, CCCP treatment could recruit PRKN onto the damaged mitochondria membrane surrounded nuclear of control oocytes, while in ZcKO oocytes, no aggregation signals were found. The results indicate that *Vps34* deletion disordered mitophagy initiation in oocytes.

### 2.5 Ablation of Vps34 Resulted in Sustained RAB7 Activation and Impaired Late Endosome Maturation

**Figure 5:**
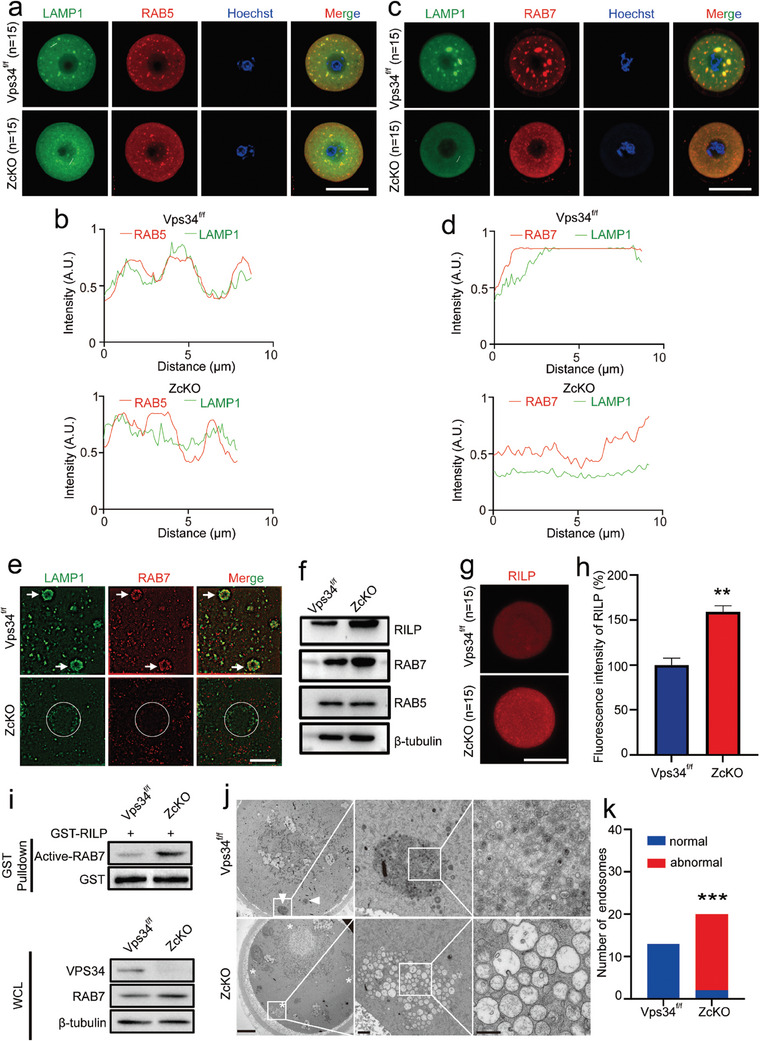
Impaired endosomal trafficking in ZcKO oocytes. GV and MII Oocytes were collected from 5 W control and ZcKO mice after superovulation. a,b) Immunofluorescent co‐staining of LAMP1 (green) with RAB5 (red) of control and ZcKO GV oocytes (a) and intensity measurement of the line shown inside the oocytes (b). c,d) Immunofluorescent co‐staining of LAMP1 (green) and RAB7 (red) (c) and intensity measurement of the line shown inside the oocytes (d). Blue, nuclear staining with Hoechst 33342; Merge, representing the superposition diagram of the fluorescence signals from the three channels. Scale bar = 50 µm. e) Ultra‐high resolution imaging of LAMP1 (green) and RAB7 (red) co‐staining in control and ZcKO MII oocytes. Scale bar = 5 µm. f) Western blots of RILP, RAB7, and RAB5 protein levels in control and ZcKO MII oocytes. GAPDH was used as an internal control. The experiment was repeated for at least three times with each run involving 80 oocytes per group. g) Immunofluorescent staining of RILP in control and ZcKO MII oocytes. h) Comparison of RILP fluorescent intensity between control and ZcKO MII oocytes. **p < 0.01. i) Western blots of whole‐cell lysates (WCL) and proteins obtained from RAB7‐RILP pull‐down assay using lysates from Vps34^f/f^ and ZcKO oocytes. j) Aggregates of late endosomes in control and ZcKO MII oocytes under TEM. White triangle, late endosomes with multi‐vesicle bodies (MVBs); white asterisk, abnormal late endosomes with enlarged vacuoles. The images from left to right are the magnifications of white frames. Scale bars = 10 µm, 1 µm, and 500 nm, respectively. k) Fisher's exact test of normal and abnormal endosomes observed under TEM in oocytes of the control and ZcKO group. ***p < 0.001.

RAB5 and RAB7 GTPases are key regulators on early to late endosome maturation and lysosome fusion. The two GTPases have also been reported to be functional in regulating mitophagy.^[^ ^40^, ^42^, ^43^ ^]^ The requirement of VPS34 phosphatidylinositol kinase activity and its product PI3P in coordination with RAB5 and RAB7 functioning on early or late endosome has been well studied. However, if VPS34 participates in regulating the early step of mitophagy remains unclear, especially when we found the failure to initiate mitophagy in CCCP treated ZcKO oocytes. We first examined the location of RAB5 and RAB7 in both control and ZcKO GV oocytes. As shown in **Figure** **5**a,b, when co‐stained with lysosome marker LAMP1, RAB5 positive early endosome was found to be present as smaller puncta near the control oocyte membrane where they showed partly overlapped with lysosomes. The RAB7 staining was shown to colocalize with LAMP1 labeled lysosome which suggested the normal endosomal trafficking process in control oocytes (Figure 5c,d). However, in ZcKO oocytes, both RAB5 and RAB7 signals were seldom colocalized with LAMP1 which the staining dispersed in the cytoplasm. Under ultrahigh resolution microscopy, we could clearly see large numbers of contacts between lysosome and late endosome (Figure 5c, arrows) in control oocytes, whereas in ZcKO oocytes, lysosomes were found in vacuoles and seldom RAB7 signals were colocalized and aggregated with LAMP1 signals (Figure 5e, circles). Western blots then demonstrated increased expression of RAB7 but not RAB5 in ZcKO oocytes (Figure 5f). Moreover, RILP (Rab Interacting Lysosomal Protein), a lysosomal protein that interacts with activated RAB7, was also shown increased expression in ZcKO oocytes (Figure 5g–i). When observing under TEM, the endosome traffic in control oocytes was manifested by the formation of late endosome with multi‐vesicle bodies (MVBs), while in ZcKO oocytes, such structure disappeared, instead, a great number of enlarged vacuoles were found (Figure 5j,k). Thus, in ZcKO oocytes, we observed the increased activity of RAB7 and the disturbance on endosome trafficking and lysosome fusion.

### 2.6 Involvement of VPS34 in Retromer Function Through Its Regulation on RAB7 Activity

**Figure 6:**
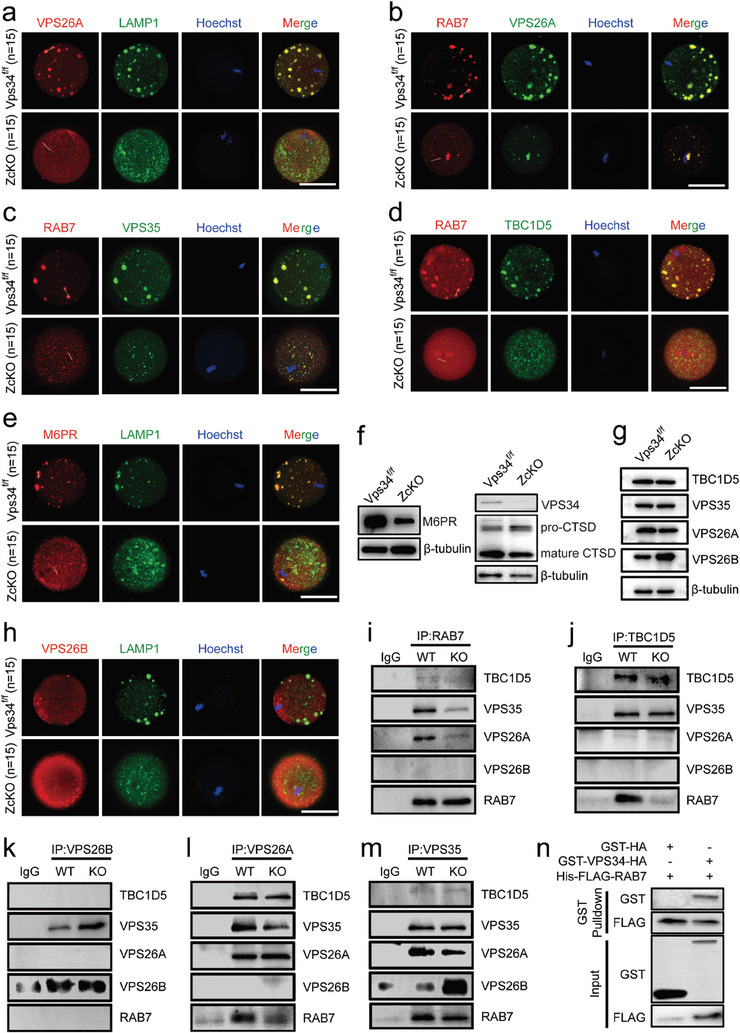
Loss of VPS34 resulted in abnormal assembly of retromer complexes in oocytes. MII oocytes were collected from 5 W control and ZcKO mice after superovulation. a‐e) Immunofluorescent co‐staining of VPS26A (red) with LAMP1 (green) (a); RAB7 (red) with VPS26A (green) (b); RAB7 (red) with VPS35 (green) (c); RAB7 (red) with TBC1D5 (green) (d), M6PR (red) with LAMP1 (green) (e) in control and ZcKO oocytes. Scale bars = 50 µm. Blue, nuclear staining with Hoechst 33342; Merge, representing a superposition of fluorescence signals from three channels. f) Western blot of VPS34 and CTSD and M6PR in control and ZcKO oocytes. β‐tubulin was used as an internal control. g) Western blots of TBC1D5, VPS35, VPS26A and VPS26B in control and ZcKO oocytes of mice. β‐tubulin was used as a loading control. h) Immunofluorescent co‐staining of VPS26B (red) with LAMP1 (green) in control and ZcKO oocytes. i–m) Interactions of RAB7 with retromer components in control and *Vps34* knockout (KO) 293T cells. *Vps34* knockout 293T cell line was constructed using the CRISPR‐Cas9 system and co‐immunoprecipitation experiments were performed as follows: RAB7 (i), TBC1D5 (j), VPS26B (k), VPS26A (l) and VPS35 (m) were used to immunoprecipitate the proteins in control and KO 293T cells, and then anti‐RAB7, anti‐TBC1D5, anti‐VPS26A, anti‐VPS26B and anti‐VPS35 antibodies were used for Western blot detection. At least 3 independent replicates were performed for each experiment. n) Direct interaction between VPS34 and RAB7 by GST pull‐down assay.

RAB7 has been reported to recruit the retromer complex onto late endosome through direct interactions with the retromer subcomplex VPS26A and VPS35.^[^ ^44^ ^]^ Besides that, retromer has also been implicated in the regulation of mitophagy through the regulation of RAB7 activity and its translocation on damaged mitochondria.^[^ ^19^ ^]^ Due to the aberrant increased activity of RAB7 and its failure to shift on mitochondria during mitophagy, we next tried to see if VPS34 function to connect the two processes through the regulation of RAB7 activity. We first examined the components of the retromer cargo recognition complex, including VPS26A and VPS35, and its associated TBC1D5, a RAB7‐specific GAP in ZcKO oocytes with co‐staining of RAB7 or LAMP1. In control oocytes, typical aggregated puncta showed the colocalization of VPS26A, VPS35 and TBC1D5 with RAB7 or LAMP1 (**Figure** **6**a–d; Figure S4d–g, Supporting Information). Moreover, we detected the localization of M6PR (Mannose‐6‐Phosphate Receptor), the best‐characterized cargo that specifically transport mannose‐6‐phosphate‐containing acid hydrolases from the Golgi complex to lysosomes, in LAMP1 positive puncta (Figure 6e; Figure S4h, Supporting Information). The staining results indicated the normal retromer function on late endosome. However, in contrast to what was observed in control oocytes, all the signals diffused in the cytoplasm, and seldom co‐staining signals were observed in ZcKO oocytes. Under higher‐resolution microscopy, the LAMP1 and VPS26A co‐staining signals could be observed in vacuoles of ZcKO oocytes, whereas M6PR signals were seldom overlapped with LAMP1 (Figure S5a,b, Supporting Information). Western blots subsequently demonstrated reduced protein levels of M6PR in ZcKO oocytes. Additionally, we discovered the transport of lysosomal enzymes, specifically the aspartyl protease cathepsin D (CTSD), was impeded in ZcKO oocytes. This was evidenced by the accumulation of pro‐CTSD and a notable decrease in mature CTSD (Figure 6f). We also conducted co‐staining experiments using the trans‐Golgi network (TGN) marker TGN46 with RAB7 and TGN46 with M6PR (Figure S5c,d, Supporting Information). The results showed that TGN was distributed around RAB7‐positive or M6PR‐positive puncta in control oocytes. However, in ZcKO oocytes, this distribution was absent. This further confirms the disturbance of retrograde trafficking after the deletion of *Vps34* in oocytes. These findings suggest the mis‐sorting of the acid hydrolases to the late endosome and a deficiency in retrograde trafficking in ZcKO oocytes.

In the study, we also identified significantly higher expression of *Vps26b* from RNA sequencing data in ZcKO oocytes. As VPS26B formed a distinct retromer with VPS35 and VPS29,^[^ ^45^ ^]^ we next tried to see the effect of VPS34 on the interactions of RAB7 with different retromer complexes. Figure 6g shows the protein levels of retromer proteins in control and ZcKO oocytes. Although the distributions of VPS26A‐retromer proteins changed a lot in ZcKO oocytes, western blot did not reveal the alterations of their protein levels. However, we observed significantly increased expression of VPS26B in ZcKO oocytes. Immunostaining then showed the distribution of VPS26B in cytoplasm and around meiotic spindle of both control and ZcKO oocytes but no co‐staining signals being observed with LAMP1 (Figure 6h). Next, in order to see the relationship between RAB7 and the two kinds of retromer complexes, we constructed a *Vps34* null 293T cell line by CRISPR‐Cas9 system and performed immunoprecipitation experiments between different components. First, we clarified the knockdown efficiency of *Vps34*, and confirmed the increased expression of VPS26B and RAB7 in the knockout group which was consistent with that in oocytes (Figure S5e, Supporting Information). Through immunoprecipitation experiments, we found decreased interactions of RAB7 with VPS26A, VPS35 (Figure 6i). When immunoprecipitation was performed with TBC1D5 antibodies, however, only the decreased expression of RAB7 was identified in VPS34‐deficient cells (Figure 6j). Moreover, VPS26B showed no bindings with RAB7, VPS26A and TBC1D5, but it revealed the significantly increased interactions with VPS35 (Figure 6k). As a result, the interactions of VPS26A with VPS35 and RAB7 reduced dramatically in KO cells (Figure 6l). After KO cell samples being immunoprecipitated with VPS35 antibody, it showed decreased expression of RAB7 and VPS26A but the expression of VPS26B heightened significantly (Figure 6m). Finally, to elucidate the role of RAB7 in VPS34‐mediated retromer function, we characterized endogenous protein interactions in 293T cells. Our results showed that VPS34 binds exclusively to RAB7, but not to any component of the VPS26A retromer complex (Figure S5f,g, Supporting Information). Additionally, Co‐IP experiments in oocytes also demonstrated the interaction between VPS34 and RAB7 (Figure S5h, Supporting Information). The direct interaction between RAB7 and VPS34 was then confirmed by GST pulldown assays (Figure 6n). These results suggest the accumulated VPS26B can competitively bind to VPS35, thereby inhibiting the formation of VPS26A‐retromer and reducing interactions between RAB7 and VPS26A‐associated TBC1D5. Consequently, the aberrant increased RAB7 activity results in deficiencies in late endosome maturation, retrograde trafficking and even mitophagy.

### 2.7 Aberrant Vesicular Trafficking in Superovulated Human Oocytes

**Figure 7:**
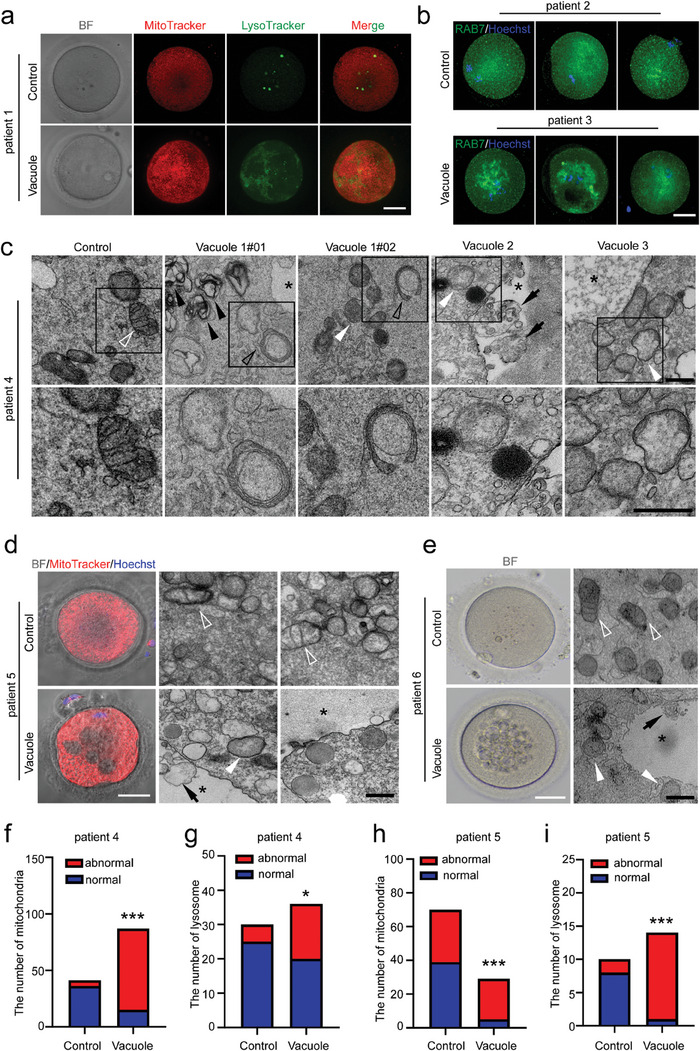
Disturbance of endosomal network in human oocytes with multiple vacuoles. a) MitoTracker (red) and LysoTracker (green) staining in MI oocytes from patient 1 with or without vacuoles. BF, bright field. Scale bar = 40 µm. b) RAB7 staining to label late endosomes in three normal oocytes from patient 2 and three vacuolated oocytes from patient 3. Scale bar = 40 µm. c‐e) TEM showed the morphology of mitochondria, lysosomes, and vacuoles in MII oocytes from patient 4, patient 5, and patient 6. One morphology was normal and three vacuolated oocytes were collected from patient 4 (c). The BF and MitoTracker staining of MII oocytes with or without vacuoles were shown in patient 5 (d). Representative multiple vacuoles under BF were shown in patient 6 (e). BF, bright field. Scale bar = 40 µm. Black asterisk, vacuoles; white hollow arrowhead, normal mitochondria; white filled arrowhead, damaged mitochondria; black hollow arrowhead, phagosomes enclosed damaged mitochondria; black filled arrowhead, accumulated secondary lysosomes; black arrows, vesicles in vacuoles. TEM, scale bars = 500 nm. f‐i) Chi‐square test of normal and abnormal numbers of mitochondria and lysosome under TEM of normal and vacuolar oocytes from patients 4 (f,g). Chi‐square test of normal and abnormal numbers of mitochondria and fisher's exact test of normal and abnormal numbers of lysosome under TEM of normal and vacuolar oocytes from patients 5 (h,i).

The desynchronization of nuclear and cytoplasmic maturation is widely acknowledged in controlled ovarian hyperstimulation for human Assisted Reproductive Technology (ART). This can be evidenced by a wide range of cytoplasmic dysmorphisms, even though meiosis can still resume.^[^ ^46^ ^]^ Several studies have reported the statistically decreased fertilization and clinical pregnancy rates in vacuolated oocytes.^[^ ^47^ ^]^ Based on the phenotype of multiple vacuoles in ZcKO oocytes and embryos, we evaluate if the dysfunctions of endosome system are involved in the vacuolization of human oocytes. Morphologically normal and vacuolated oocytes from the same patient were collected for comparison. In particular, our findings revealed the aggregation of mitochondria and the disappearance of mature lysosomes in vacuolated oocytes (Patient 1, **Figure** **7**a). In normal oocytes, late endosomes (RAB7‐positive puncta) were uniformly distributed throughout the cytoplasm (Patient 2, Figure 7b), while in vacuolated oocytes, the aggregated RAB7 signals were observed (Patient 3, Figure 7b). Under TEM observation, it's common to find numerous swollen, vacuolated or cristae disappeared mitochondria scattered in the cytoplasm of vacuolated oocytes (Patient 4, Patient 5, and Patient 6, Figure 7c–e). Sometimes, we also observed phagosomes engulfing damaged mitochondria (Patient 4, Figure 7c: Vacuole 1 oocyte). Moreover, in the vacuole region (* labeled area), we identified vesicles containing floccular contents or vesicles enclosing smaller vesicles (black arrows, Figure 7c–e). Normal and abnormal mitochondria and lysosomes were then counted in the electron micrographs of non‐vacuolated and vacuolated oocytes from Patient 4 (Figure 7f,g) and Patient 5 (Figure 7h,i). Our findings indicate that vacuolated oocytes exhibited a significantly higher number of abnormal mitochondria and lysosomes compared to their non‐vacuolated counterparts. These results suggest dysfunctions of organelles, including mitochondria, lysosome, or endosome system in vacuolated human oocytes.

### 2.8 Activation of VPS34 Improved Oocyte Quality in Aged Mice

**Figure 8:**
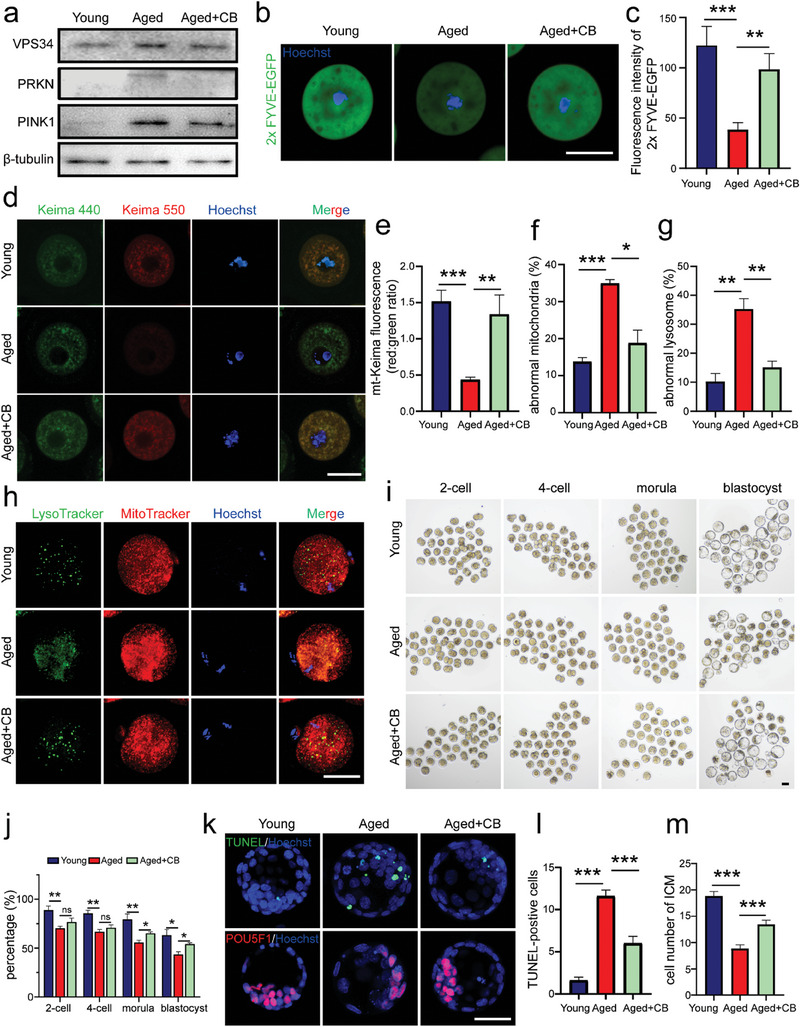
Improvement of oocyte quality after VPS34 activation in aged mice. Female mice at 4 W and 10 M of age were injected with PMSG for GV oocytes or COC (cumulus‐oocyte complex) collection. Isolated GV oocytes were microinjected with 2×FYVE‐EGFP mRNA, mt‐Keima or just cultured directly in 2 µM milrinone and incubated with or without 20 µM Corynoxine B (CB) for 16 h. a) Western blot of VPS34, PRKN, PINK1 in GV oocytes of each group. The expression of β‐tubulin was used as the internal control. b) The fluorescence of 2×FYVE‐EGFP in each group. Green, 2×FYVE‐EGFP; Blue, Hoechst 33342. Scale bar = 50 µm. c) Comparison of 2×FYVE‐EGFP fluorescence intensity in oocytes of each group (n = 15 oocytes). d) Mitophagy flux in GV oocytes of each group after microinjection with mt‐Keima mRNAs. Images of oocytes were collected at fluorescent emission 550 nm (red) and 440 nm (green), respectively. Scale bar = 50 µm. e) Measurement of mitophagy levels determined by the ratio of fluorescent density 550 (red)/440 (green) per oocyte. n = 5 oocytes in each group. f‐h) Evaluation of mitochondria and lysosomes in MII oocytes of each group. COC were cultured in IVM media and treated with or without CB for 12 h. Obtained MII oocytes were then stained with MitoTracker (red) and LysoTracker (green) (h). The percentages of oocytes with mitochondria aggregations (f) or with few lysosomes (g) were calculated. The experiment were repeated for three times with total 98 oocytes in young, 106 oocytes in aged and 93 oocytes in aged+CB groups. Scale bar = 50 µm. i,j) Early embryonic development in each group. MII oocytes obtained from cultured COC of each group were used for IVF and embryonic development was followed until blastocyst stage (i). The percentage of embryonic development at 2‐cell, 4‐cell, morula, and blastocyst stage in young (n = 160 embryos), aged (n = 154 embryos), and aged+CB (n = 161 embryos) group (j). The experiment was repeated four times. Scale bar = 50 µm. k) TUNEL (green) and POU5F1 (red) staining of blastocysts collected from each group. Upper panel, TUNEL staining; lower panel, POU5F1 staining to label inner cell mass (ICM). Scale bar = 50 µm. l,m) The number of TUNEL‐positive cells (green) (l) and ICM cell numbers (red) (m) in every blastocyst of the groups (n = 15). All graphs are presented as the means ± SEM. *p < 0.05, **p < 0.01, ***p < 0.001. ns = no significance.

Unlike what was observed in human oocytes, it's seldom to observe vacuolation in mouse oocytes after superovulation treatment. However, it's reported that mice exposed to repeated superovulation showed deteriorated oocyte quality.^[^ ^48^ ^]^ Our previous study demonstrated the blockage of mitophagy in aged oocytes led to the accumulation of mitochondria damage and the defects on oocyte meiosis.^[^ ^25^ ^]^ We then collected GV oocytes from young and aged female mice to evaluate the effect of VPS34 on oocyte quality during the aging process. From Western blot results, the increased protein levels of VPS34 were observed in aged oocytes (**Figure** **8**a). Given that the phosphatidylinositol kinase VPS34 phosphorylates PI to produce PI3P and subsequently recruits proteins containing the FYVE structural domain,^[^ ^49^, ^50^ ^]^ we microinjected 2×FYVE‐EGFP mRNA to measure PI3P levels in oocytes. Contrary to the elevated protein levels of VPS34, we found a significant decrease of PI3P production in aged oocytes based on the fluorescence density. To investigate if VPS34 activity controls oocyte quality during the aging process, we treated GV oocytes from older mice with the VPS34 activator, corynoxin B (CB).^[^ ^51^ ^]^ Figure 8b,c showed the elevated VPS34 activity, evident through the increased expression of FYVE‐GFP in CB‐treated oocytes. Furthermore, CB treatment restored the blocked mitophagy flux in aged oocytes as shown by decreased expression of PRKN and PINK1 and increased colocalization of mt‐Keima red and green fluorescent signals (Figure 8d,e). We then collected cumulus‐oocyte complexes (COCs) from both young and old mice, treating the aged COCs with or without CB for IVM and IVF. MitoTracker and LysoTracker staining demonstrated that CB treatment improved mitochondrial and lysosomal functions in aged oocytes (Figure 8f–h). Additionally, IVF results showed increased embryonic development rates (Figure 8i,j). TUNEL and POU5F1 staining indicated reduced cell apoptosis and an increased number of ICM (inner cell mass) cells in blastocysts derived from oocytes collected from CB‐treated COCs (Figure 8k–m). These findings underscore the necessity of VPS34 activity in maintaining the mitophagy flux in GV oocytes, suggesting a correlation between VPS34 activity and oocyte aging. Activation of VPS34 by CB treatment can restore mito/autophagy flux in aged oocyte and ultimately improve the developmental competency of the resulting embryos.

## 3 Discussion

In mammals, the early embryonic development is initiated by the degradation of maternally inherited mRNAs, proteins, and other metabolites. These events are important for ZGA because before that the embryos are transcriptionally silent.^[^ ^52^ ^]^ Until now, three major pathways are reported for the degradation of maternal proteins: ubiquitin‐proteasome pathway (UPS), autophagy, and endocytosis.^[^ ^53^ ^]^ Both UPS and endocytosis are involved in the degradation of selected proteins, whereas autophagy refers as the dynamic protein turnover which includes not only the degradation of unnecessary proteins, but also the recycling of amino acids for de novo protein synthesis.^[^ ^21^, ^54^ ^]^ In the study, the blockage of autophagy flux was manifested in *Vps34* deleted oocytes and derived embryos by using VPS34‐GFP‐LC3 transgenic mice, BafA1 treatment as well as the increased expression of autophagy‐related p62 and LC3B I/II proteins. The embryonic development derived from ZcKO oocytes was halted at the 2‐ to 4‐cell stage. This phenotype is similar to, but more severe than what was reported about the *Atg5* null embryos which exhibit developmental arrest at 4‐ to 8‐cell stage. This means the maternal effect of VPS34 on early embryonic development. Actually, from the RNA sequencing data, large amounts of DEGs expressed in ZcKO 2‐cell embryos were related with maternal mRNA decay, ZGA, and 2‐cell transition. Moreover, like what was overserved in *Atg5* null embryos, we also found significantly decreased protein synthesis in ZcKO 2‐cell embryos. Autophagy has been reported to be involved in maternal mRNA degradation.^[^ ^55^ ^]^ Thus, the failure of ZGA and significantly decreased new protein synthesis in ZcKO mice may lead to the arrest of embryonic development at 4‐cell stage. However, in contrast to the extremely low level of translation, we found an aberrant activation of mTOR signaling pathway in ZcKO 2‐cell embryos. VPS34 has been proposed as a nutrient/amino acid sensitive regulator of mTORC1 activity.^[^ ^56^ ^]^ In the absence of VPS34, although amino acid‐ or insulin‐stimulated mTOR activation was suppressed, the steady‐state level of mTOR signaling remained unaffected.^[^ ^26^ ^]^ This is consistent to the unaffected mTOR signaling in MII ZcKO oocytes, but contrasts with our findings in ZcKO 2‐cell embryos. The interrelationship between mTORC1 and autophagy in maintaining nutrient homeostasis has been reported to be governed by a core set of proteins sensing nutrients at lysosomal membranes.^[^ ^57^ ^]^ Hyperactivation of MTORC1 has been shown to cause lysosomal dysfunction by negatively regulating the fusion of the autophagosome with lysosomes through interacting and phosphorylation of UVRAG.^[^ ^58^ ^]^ Thus, the hyperactivation of mTOR signaling pathway may contribute to the blockage of autophagy flux in 2‐cell embryos derived from ZcKO mice. Meanwhile, we also observed the increased transcription as shown by EdU labeling in ZcKO 2‐cell embryos. VPS34 has been shown to mediate the nuclear translocation of EGFR.^[^ ^59^ ^]^ It also mediates membrane transport from the outer nuclear membrane toward the cell periphery in *Caenorhabditis elegans*.^[^ ^60^ ^]^ We suppose this may represent a compensatory response due to defects on VPS34 mediated membrane transport system in oocyte.

Due to the property of maternal inheritance of mitochondria in development, the quantity and quality of oocyte mitochondria are key determinants of oocyte competence for fertilization and early embryonic development. Mitophagy, a specialized autophagy that degrades damaged mitochondria, has been implicated its importance in oocyte maturation and oocyte quality control during ovarian aging.^[^ ^25^, ^61^ ^]^ In the study, we found vast damaged mitochondria accumulated in ZcKO oocytes which was shown by abnormal mitochondria distributions and impaired mitochondria functions. Since the first few days of embryonic development depend on the ATP provided by the mitochondria stored in oocyte,^[^ ^62^, ^63^ ^]^ the extremely low level of ATP in ZcKO oocytes and embryos may contribute to the arrest of embryonic development. The blockage of mitophagy in ZcKO oocytes was then manifested by Mito‐Keima labeling, the aberrant co‐staining signals of PINK1/LAMP1 and ATG9/LAMP1. Moreover, when GV oocytes were treated with CCCP to induce mitophagy, we found the failure for PRKN recruitment on damaged mitochondria. Upon mitochondria damage, activation of PINK1 on mitochondria outer membrane stimulates the recruitment of PRKN, a cytosol E3 ubiquitin ligase, to the depolarized mitochondria and induces a cumulative ubiquitination and the formation of ubiquitin chains on multiple proteins. PINK1 and the resulted ubiquitin signals recruit autophagy receptors, NDP52 and POTN and then binds to LC3B facilitating the engagement of mitophagosome formation with the help of ATG9‐containing vesicles.^[^ ^64^ ^]^ Therefore, in addition to the previously established role of VPS34 in the fusion of mitophagosome with lysosome, our results reveal for the first time the involvement of VPS34 in the processes of mitophagy initiation and mitophagosome formation.

RAB7 is one of the most studied Rab proteins which controls maturation of late endosome, transport from late endosome to lysosome, fusion of late endosome with lysosome and other physiological processes such as phagocytosis, mitophagy or retrograde trafficking.^[^ ^19^, ^65^ ^]^ In the study, the association of VPS34 and RAB7 was first demonstrated by the increased protein levels and hyperactivation of RAB7 in ZcKO oocytes. The defects of late endosome trafficking were then manifested by the failure to form endolysosomes and the phenotype of vacuoles in ZcKO oocytes and derived embryos. This is consistent with what was observed in *Vps34* ^−/−^ mouse embryonic fibroblasts (MEFs) in which the elevated RAB7‐GTP levels stemmed from the failure of VPS34 to recruit the TBC1D2 family of GAPs.^[^ ^66^ ^]^ However, different from the rescuing effect of RAB7 silencing or TBC1D2 overexpression in *Vps34*‐deficient cells, we couldn't find such effect in oocytes by microinjection of *Rab7* siRNA and Rab7^T22N^, which represents inactive RAB7‐GDP binding status.^[^ ^25^ ^]^ Moreover, microinjecting *Vps34* mRNA in knockout zygote only obtained a minimal recovery of embryonic development. The findings suggest that other than endosomal trafficking, VPS34 plays multiple roles in regulating oocyte cytoplasmic maturation.

Recent studies revealed the role of RAB7 on mitophagosome formation by recruiting ATG9‐bearing vesicles around damaged mitochondria. This process relies on an intact Rab cycling process mediated by RAB7‐specific GEF and GAPs and is also dependent on the retromer complex.^[^ ^25^, ^67^ ^]^ Loss of retromer complex subunits VPS29, VPS35 or RAB7 specific GAP, TBC1D5 in 293T cells, all resulted in the heightened RAB7 activity and the failure of its translocation on damaged mitochondria after CCCP treatment.^[^ ^19^ ^]^ However, activating RAB7 is also required for the retromer‐dependent membrane recruitment because inhibition of TBC1D5 can enhance the function of retromer.^[^ ^68^ ^]^ Thus, RAB7 is differentially regulated according to its functions on retrograde trafficking and mitophagy. In the study, deletion of *Vps34* in oocyte showed the impairment on both the retrograde trafficking and mitophagy. We then demonstrated the reduced interactions between RAB7 and the retromer complex subunits, VPS26A, VPS35 and TBC1D5. This can be explained by the failure of membrane recruitment of retromer complex, and the impaired retrograde trafficking of acid hydrolyses to lysosomes. Furthermore, our study revealed a previously unknown mechanism for the regulation of RAB7 activity via another retromer complex containing VPS26B. We found the increased expression of VPS26B in ZcKO oocytes which will compete with its paralogous VPS26A to form another retromer complex and then reduced the interactions between RAB7 and TBC1D5‐VPS26A retromer complex. The decrease in VPS26A retromer complex led to dysfunctional retrograde trafficking, while increased RAB7 activity was associated with mislocation of RAB7, the failures on late endosome formation, initiation of mitophagy, and the formation and maturation of mitophagosome and autophagosome. VPS26A‐ and VPS26B‐retromer are functional distinct retromer complex.^[^ ^45^ ^]^ It's noteworthy that RAB7 only showed the interactions with TBC1D5‐VPS26A retromer, but not with VPS26B. Until now, it's still not clear the functions of VPS26B‐retromer and its regulatory mechanisms. Our study further enhances our understanding of the essential role of the VPS34 complex in conjunction with RAB7 in regulating various forms of vesicular trafficking within the cell.

In human ART, compromised oocyte quality induced by ovarian stimulation can be reflected as a great variety of abnormal cytoplasmic morphologies, such as vacuoles, refractile bodies, dark/brown color of the cytoplasm or aggregates of smooth endoplasmic reticulum (SER) et al. Vacuolization in cytoplasm of human oocytes accounts for an incidence rate of only 4%, but it is associated with poor clinical outcomes and shows negative effects on blastocyst formation.^[^ ^5^ ^]^ This is dependent on the size and number of vacuoles. As for the origin of these vacuoles, it was hypothesized as the fusion of smaller vesicles derived from the endoplasmic reticulum or the Golgi apparatus. In eukaryotic cells, vesicle trafficking is the fundamental process to maintain the homeostasis of membrane‐enclosed organelles in eukaryotic cells.^[^ ^69^ ^]^ Besides endoplasmic reticulum (ER) and Golgi apparatus, endosomes, multivesicular body, lysosome or plasma membrane are also well‐characterized vesicle trafficking organelles. From TEM analysis, LysoTracker or RAB7 staining in human vacuolated oocytes, we found the vacuolated defects related with endo‐lysosome trafficking pathway. Meanwhile, we also noticed large amounts of damaged mitochondria accumulated in cytoplasm. This further supports our findings in ZcKO mice, highlighting the coordinated action between RAB7 and VPS34 in regulating multiple vesicle trafficking during oocyte cytoplasm maturation. Different from what was observed in humans, cytoplasmic vacuoles were hardly detected in mouse oocyte under physiological conditions. However, the PI3P production as shown by FYVE expression decreased significantly in mouse aged oocytes. Moreover, after the aged oocytes being treated with VPS34 activator CB, we found the elevated FYVE expression levels and the restored mitophagic flux. The improved oocyte quality was then manifested by the recovery of mitochondria and lysosome functions and increased embryonic development after IVM and IVF experiments. Until now, many cytoplasmic processes related with oocyte competence have remained elusive. Our study has shed light on the significance of VPS34‐mediated multiple vesicle‐trafficking pathways in determining the oocyte competence. The levels of oocyte PIP3 may serve as a reliable marker for assessing oocyte quality. Activating VPS34 could potentially enhance the success rates of human ART.

**Figure 9:**
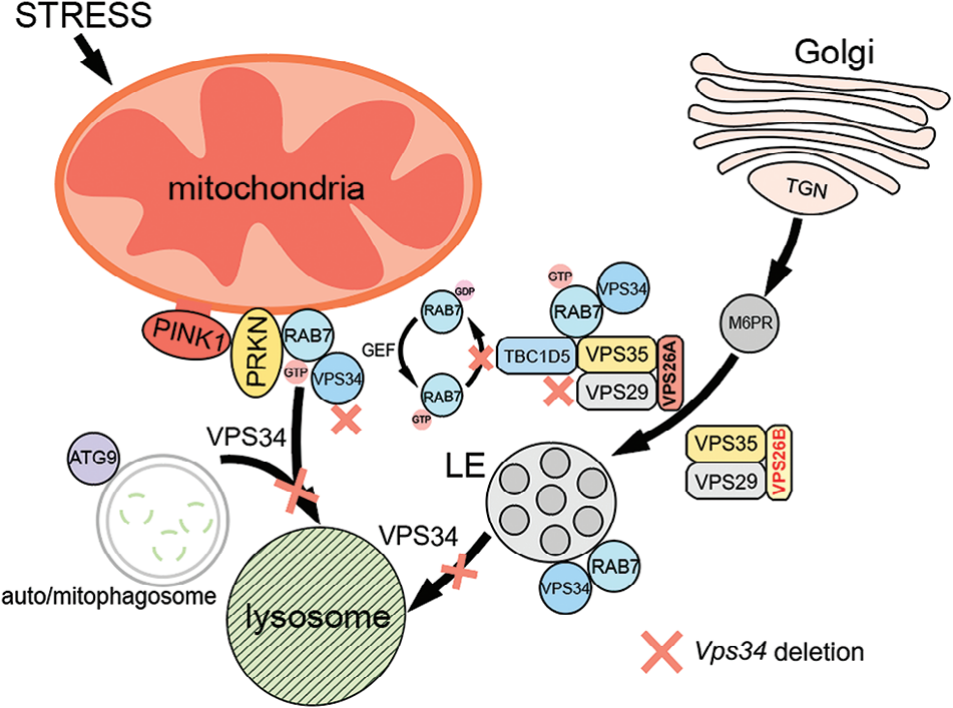
A schematic diagram illustrates the functional mechanism of VPS34 in oocytes. The critical role of VPS34 in oocyte is manifested by the blockage of mitophagy and autophagy flux in both ZcKO oocytes and derived embryos. VPS34 is also crucial for regulating RAB7 activity and facilitating its translocation on mitochondrial membrane during mitophagy initiation, as well as for the RAB7‐mediated ATG9 trafficking during mito/autophagosome formation. The RAB7 GAP TBC1D5, in complex with VPS26A retromer, regulates the activity of RAB7 by converting it from GTP to GDP state. The regulatory process is reliant on the direct interaction between RAB7 and VPS34. When VPS34 is deleted, it leads to an increased expression of VPS26B. VPS26B then competes with VPS26A to form an alternative retromer complex, which consequently reduces the interactions between TBC1D5 and RAB7. As a result, there is an increase in RAB7 activity. When RAB7 is hyperactivated, it is unable to properly translocate to the mitochondria membrane, leading to a failure in forming ATG9‐mediated mito/autophagosome formation during PRKN‐mediated mitophagy. Thus, VPS34 is not only involved in the maturation of late endosomes and lysosomes but also plays a crucial role in initiating mitophagy by regulating the activity and subcellular location of RAB7. In ZcKO oocytes, the accumulation of mitochondrial damage and blockage of autophagy ultimately result in impaired oocyte developmental competence.

In summary, the oocyte specific function of VPS34 was delineated by conditional knockout of *Vps34* in growing oocyte. Our results revealed the central role of VPS34 in regulating a series of vesicular trafficking processes occurred in cytoplasm during oocyte maturation. We also elucidate a new mechanism about the regulation of VPS34 on mitophagy and retromer trafficking through the regulation on RAB7 activity and its subcellular locations (**Figure** **9**). Defects in vesicle trafficking pathways and mitochondria damage were subsequently observed in both human ovulated oocytes and oocytes from aged mice. Notably, we observed enhanced embryonic development when VPS34 was activated in aged mouse oocytes. These findings present a promising approach to improve oocyte quality in human ART and enhance fertility, particularly for those women with advanced maternal age.

## 4 Experimental Section

### Human Oocyte Collection and Ethical Approval

The human oocytes were donated by patients undergoing assisted reproduction treatment at the First Affiliated Hospital of Nanjing Medical University after signing a written informed consent form, and the use of these human oocytes was clearly explained to the patients. The Ethics Committee of the First Affiliated Hospital of Nanjing Medical University approved this study (research license: 2012‐SR‐128, 2023‐SZ‐01). Control oocytes and vacuolar oocytes used in each experiment were from the same patient as far as possible.

### Mice

*Vps34* ^flox/flox^ (Vps34^f/f^) mice were purchased from The Jackson Laboratory (019081), *Zp3‐Cre* mice were obtained from the Model Animal Research Center of Nanjing University (Nanjing, China). These mice were maintained on identical C57BL/6J genetic backgrounds. The oocyte‐specific *Vps34* knockout mice were created by a breeding scheme of Vps34^f/f^ females crossing with Vps34^f/f^; *Zp3‐Cre* males (*Zp3‐Cre*/+; Vps34^f/f^, ZcKO mice). To create ZcKO mice with a GFP tag on the LC3 protein, we first crossed GFP‐LC3#53 (no. 00806) mice with Vps34^f/f^ male mice to generate GFP‐LC3; Vps34^f/+^ mice.^[^ ^70^, ^71^ ^]^ Next, GFP‐LC3; Vps34^f/+^ male and female mice were mated to generate GFP‐LC3; Vps34^f/f^ mice. GFP‐LC3; Vps34^f/f^ females and *Zp3*‐*Cre*/+ males were crossed to produce GFP‐LC3; *Zp3‐Cre*/+; Vps34^f/f^ (GFP‐LC3‐ZcKO) females for subsequent experiments. All animal procedures and protocols were approved by the Animal Care and Use Committee at Nanjing Medical University and were conducted in accordance with the institutional guides for the care and use of laboratory animals. Fertility tests were carried out by mating 6‐week‐old Vps34^f/f^ and ZcKO females with normal adult C57BL/6J males for 6 months. The number of pups for each litter was recorded at birth, and the average number of pups per litter was calculated at the end of the testing period.

### Oocyte Collection and Culture

For collecting GV oocytes, mice were injected with 5 IU of pregnant mare serum gonadotropin (PMSG, Ningbo Sansheng Pharmaceutical Co., Ltd., China) at 48 h earlier; the ovaries were removed in M16 medium (Sigma‐Aldrich, USA) supplemented with 2.5 µM milrinone (Sigma–Aldrich, 78415‐72‐2) to maintain meiotic arrest. GV‐stage oocytes were collected by puncturing large follicles with needles, and cultured in M16 medium (Sigma‐Aldrich, USA) under mineral oil (Sigma‐Aldrich, USA) at 37 °C and 5% CO_2_ in a humidified incubator.

For obtaining MII oocytes, mice were superovulated by injecting 5 IU of PMSG, followed by 5 IU of human chorionic gonadotropin (hCG, Ningbo Sansheng Pharmaceutical Co., Ltd., China) after 48 h. Then, the mice were sacrificed at 14 h after the hCG treatment, and cumulus cell‐oocyte complexes were recovered from oviductal ampullae. After a treatment with hyaluronidase (1 mg mL^−1^) in M2 medium to free the cumulus cells, the oocytes were collected.

### In Vitro Fertilization (IVF)

4‐week‐old and 10‐month‐old female mice were injected with PMSG and 48 h later, cumulus‐oocyte complexes (COCs) were isolated from their ovaries. These COCs were then cultured for 16 h at 37 °C with 5% CO_2_ in MEMα medium containing EGF (Sigma‐Aldrich, SRP3196), 0.3% BSA (Sigma‐Aldrich, A7906), and 10% FBS. The cauda epididymis from 10‐week‐old male mice were isolated, and sperm were transferred into HTF medium (Sigma‐Aldrich, MR070) with 10% FBS (SORFA, SX1111) for 1 h at 37 °C with 5% CO_2_ to facilitate sperm capacitation. COCs were incubated in 200 µL HTF medium with capacitated sperm for in vitro fertilization. Following 6 h of incubation, the fertilized oocytes were transferred to KSOM medium (Sigma‐Aldrich, MR‐106) and maintained at 37 °C to monitor embryonic development at 24, 48, 72, and 96 h, respectively. Subsequently, blastocysts from each group were fixed in 4% PFA for TUNEL and POU5F1 staining, allowing the assessment of apoptosis and the cell count of ICM in each embryo.

### Mouse Embryo Collection and Cultivation

Female mice were superovulated with an intraperitoneal (ip) injection of 5 IU PMSG followed by 5 IU hCG 46–48 h later, and mated with male mice at a ratio of 1:1. Mating was confirmed by identification of a vaginal plug the next morning. Mice were killed at post‐hCG 27 h. Zygotes were obtained by flushing the excised oviduct with M2 media and cultured in 35 mm petri dishes (JET) in 30–50 µL drops of KSOM (Millipore) under 3 mL mineral oil (Thermo Fisher Scientific) in 5% CO_2_ at 37 °C in a humidified incubator. Continuous observations were recorded in the subsequent development.

### Real‐Time PCR Analyses

MII oocytes or Two‐cell embryos were collected and total RNA was isolated by RNAprep Pure Micro Kit (DP420, TIANGEN, Beijing, China) according to the manufacturer's instruction. After reverse‐transcription, Eva Green‐based qPCR was performed using SsoFast EvaGreen Supermix With Low ROX (Bio‐Rad Laboratories, PN 172–5211) on an ABI StepOnePlus platform (Thermo Fisher Scientific, Waltham, USA). The ΔΔCt method was used to quantify various mRNAs with the GAPDH amplification signal as the internal control. The specificity of the PCR products was assessed by melting curve analyses. All Primer sequences were as follows: Vps34#F, 5ʹ‐CCTGGACATCAACGTGCAG‐3ʹ, 5ʹ‐ TGTCTCTTGGTATAGCCCAGAAA‐3ʹ; Vps26b#F, 5ʹ‐CTGAATGATGCGGAGAGTAGGA‐3ʹ; Vps26b#R, 5ʹ‐ TAGGCTCACTTTCCCCGAGAC‐3ʹ; Ipo11#F, 5ʹ‐GCCACCAGTCAGGATACTGC‐3ʹ; Ipo11#R, 5ʹ‐ CAACACTGAATAGAAACCTGGCT‐3ʹ; Ndufv#F, 5ʹ‐TTTCTCGGCGGGTTGGTTC‐3ʹ; Ndufv#R, 5ʹ‐ GGTTGGTAAAGATCCGGTCTTC‐3ʹ; Slc25a31#F, 5ʹ‐ATGTCGAACGAATCCTCCAAGA‐3ʹ; Slc25a31#R, 5ʹ‐AGCTTCACACGCTCGATGG‐3ʹ; Slc2a8#F, 5ʹ‐CCCTTCGTGACTGGCTTTG‐3ʹ; Slc2a8#R, 5ʹ‐TGGGTAGGCGATTTCCGAGAT‐3ʹ; Wdr45b#F, 5ʹ‐GCCATTCACTATCAATGCACATC‐3ʹ; Wdr45b#R, 5ʹ‐ TCGTGGCTGAAGTTAATGCAG‐3ʹ; Ppp1ca#F, 5ʹ‐ATGTCCGACAGCGAGAAGC‐3ʹ, 5ʹ‐ACAGCCGTAGAAGGTCATAGT‐3ʹ; Gabra1#F, 5ʹ‐AAAAGTCGGGGTCTCTCTGAC‐3ʹ, 5ʹ‐CAGTCGGTCCAAAATTCTTGTGA‐3ʹ; Nsun5#F, 5ʹ‐CTGAAGCAGTTGTACGCTCTG‐3ʹ, 5ʹ‐CCCTTCCCCAGCAATAATTCAT‐3ʹ; Gprasp2#F, 5ʹ‐ATGGGTTCTTGGTGCTATCCC‐3ʹ, 5ʹ‐ CCTGGACCTAGTGTTCACCTC‐3ʹ.

### Histological Analysis and Quantification of Ovarian Follicles

Ovaries used for histological analysis were collected from adult female mice, fixed in 4% paraformaldehyde (PFA) (pH 7.5) overnight at 4 °C, dehydrated, and embedded in paraffin. Paraffin‐embedded ovaries were sectioned serially at 5 µm thickness for hematoxylin and eosin (H&E) staining. One or both ovaries from more than three mice of each genotype were used for analysis. Ovarian follicles were quantified as previously described^[^ ^72^ ^]^; paraffin‐embedded ovaries were serially sectioned at 5 µm thickness, and every section was mounted on slides in turn. Then, these sections were stained with H&E for morphological analysis. Ovarian follicles at different developmental stages, including primordial and activated follicles, were counted in collected sections of an ovary based on the accepted standards established by Pedersen and Peters.^[^ ^73^ ^]^ In each section, only follicles in which the nucleus of the oocyte was clearly visible were scored.

### Immunofluorescence Staining

Mice were killed at 14‐16 h post‐hCG administration to obtain the MII stage of oocytes by flushing the excised oviduct with M2 media. Oocytes were placed in M2 media containing 1 mg mL^−1^ hyaluronidase to remove the cumulus cells. These oocytes were then placed in Tyrode's buffer (Sigma‐Aldrich, T1788, pH 2.5) to remove the zona pellucid (ZP), fixed in 4% PFA solution for 30 min at room temperature (RT), and permeabilized in 0.25% TritonX‐100 for 20 min at RT. Every step above was followed with the oocytes being washed with 0.1% bovine serum albumin‐phosphate buffer saline (BSA‐PBS) for three times. After that, oocytes were preincubated in 1% BSA‐PBS for 60 min at RT, and incubated with primary antibodies overnight at 4 °C. Then oocytes were incubated in fluorescent‐labeled secondary antibodies (1:1000 dilution) and subsequently incubated in Hoechst 33342 (1:1000 dilution) for 60 and 15 min at RT without ambient light, respectively. Finally, oocytes were imaged with a ZEISS LSM700 laser scanning confocal microscope or a NIKON N‐SIM Super‐resolution microscopy. Primary and secondary antibodies were obtained from following commercial sources: rabbit monoclonal anti‐VPS34 (Cell Signaling Technology, 4263), rat monoclonal anti‐LAMP1 (DSHB, 1D4B), rabbit monoclonal anti‐α‐tubulin (Cell Signaling Technology, 2125), rabbit monoclonal anti‐PINK1 (Abcam, ab216144), rabbit polyclonal anti‐ATG9 (ZEN BIO, 381260), mouse monoclonal anti‐RAB7 (Cell Signaling Technology, 95746), rabbit polyclonal anti‐PRKN (ZEN BIO, 3381626), mouse monoclonal anti‐TOM20 (Abcam, ab283317), rabbit monoclonal anti‐RAB5 (Cell Signaling Technology, 3547), rabbit monoclonal anti‐RILP (Abcam, ab302492), rabbit monoclonal anti‐VPS26A (Cell Signaling Technology, 99384), rabbit monoclonal anti‐RAB7 (Cell Signaling Technology, 9367), rabbit polyclonal anti‐TBC1D5 (Proteintech, 17078‐1‐AP), rabbit monoclonal anti‐VPS35 (Cell Signaling Technology, 81453), rabbit monoclonal anti‐M6PR (Cell Signaling Technology, 14364), rabbit polyclonal anti‐VPS26B (affinity biosciencs, DF4606), rabbit polyclonal anti‐POU5F1 (Abcam, ab230429), rabbit monoclonal anti‐TOM20 (Cell Signaling Technology, 42406), Alexa Fluor 488 donkey anti‐mouse IgG (Thermo Fisher Scientific, A21202), Alexa Fluor 594 donkey anti‐mouse IgG (Thermo Fisher Scientific, A21203), Alexa Fluor 488 donkey anti‐rat IgG (Thermo Fisher Scientific, A21208), Alexa Fluor 594 donkey anti‐rabbit IgG (Thermo Fisher Scientific, A21207), Alexa Fluor 488 donkey anti‐rabbit IgG (Thermo Fisher Scientific, A32790), Alexa Fluor 647 goat anti‐ rabbit IgG (Thermo Fisher Scientific, A31573). For TUNEL staining, blastocysts were labeled with TUNEL Apoptosis Detection Kit (YEASEN, 40307ES60) and the TUNEL‐positive cells were counted in each blastocyst.

### Quantification of ROS

Oxidation‐sensitive fluorescent probe (dichlorofluorescein, DCFH) was used to determine the quantity of ROS production. Oocytes were loaded with the probe by 30 min incubation at 37 °C in M2 medium (Sigma–Aldrich, USA) supplemented with 10 µM of DCFH diacetate (DCFH‐DA) (Yeasen). After being washed thrice in M2 medium supplemented with bovine serum albumin (BSA), oocytes were placed M2 medium under mineral oil in a glass bottom petri dish (NEST Biotechnology, 801002). Fluorescence was examined by laser scanning microscopy using a ZEISS LSM 700 laser scanning confocal microscope.

### Characterization of Mitochondrial Parameters or Lysosome Tracers

For mitochondrial staining or inner mitochondrial membrane potential staining, live denuded oocytes were cultured in M2 medium containing 1 µM MitoTracker Red (Yeasen) or 2 µM JC‐1 fluorochrome (Yeasen) for 30 min at 37 °C under 5% CO2 in a humidified incubator. For lysosomal tracing, live denuded oocytes were incubated for 30 min at 37 °C under 5% CO2 with 1 µM LysoTracker (Yeasen) working solution. All staining working solutions were prepared in advance and preheated with mineral oil for at least 15 min. After incubation, oocytes were cleaned with preheated M2 medium before image acquisition. Images were captured by laser scanning confocal microscopy.

### Chromosome Spread

MII oocytes were cultured in M2 medium for 10 min after removal of zona pellucida in Tyrode's buffer and then treated with 1% sodium citrate for 60 s. The oocytes were then fixed on glass slides in 1% PFA solution containing 0.15% TritonX‐100 and air dried at room temperature. The samples were washed three times with 0.1% BSA‐PBS, and then blocked with 1% BSA‐PBS for 1 h at room temperature. Chromosomes were stained with Hoechst 33342 and observed under a laser scanning confocal microscope.

### Protein Synthesis Assay

2‐cell embryos were incubated with G‐1 (Vitrolife) diluted HPG working solution for 20 min at 37 °C. The culture medium was removed, and the embryos were washed three times with G‐MOPS (Vitrolife), then fixed with 4% PFA for 60 min at room temperature, washed three times with 0.1% BSA‐PBS for 5 min each, and then permeabilization with 0.5% Triton‐X100 for 20 min at room temperature. After washing with 0.1% BSA‐PBS for 3 times, add 1X HPG staining system (prepared according to the manufacturer's instructions) (Thermo Fisher Scientific, C10428), incubate at room temperature from light for 50 min, then wash with 1% BSA‐PBS for 3 times, and stain the nucleus with Hoechst 33342. After washing three times, the embryos were loaded in quench‐resistant microdrops on the slide containing poly‐lysine, and the slides were sealed with cover glass. Signals were detected using a laser‐scanning confocal microscope.

### Western Blotting

The oocytes were washed with cold PBS, then added to RIPA lysate (Solarbio, R0010) containing protease inhibitor (Solarbio, K106993P), frozen and thawed twice with liquid nitrogen, and boiled for 5 min after adding loading buffer. Cultured cells were first washed 2–3 times with cold PBS, then lysed using lysate containing protease inhibitors, broken by sonication, supernatant collected, loading buffer added, and boiled for 5 min. The protein samples were electrophoresed on 10% SDS‐PAGE gel and transferred to PVDF (Thermo Fisher Scientific, 88518) membrane. The cells were blocked with 5% low‐fat dry milk diluted with PBST for 1 h at room temperature and incubated with suitable primary antibodies overnight at 4 °C. After three washes of TBST, samples were incubated with HRP‐conjugated secondary antibodies. Signals were detected using the ECL Plus Western Blotting detection system (Beyotime, China). Tubulin was used as a loading control. Primary and secondary antibodies were obtained from following commercial sources: rabbit monoclonal anti‐CSTD (Abcam, ab305062), rabbit monoclonal anti‐LC3B (Cell Signaling Technology, 3868), mouse monoclonal anti‐p62 (Cell Signaling Technology, 88588), mouse monoclonal anti‐GAPDH (ZEN BIO, 200304–7E4), mouse monoclonal anti‐β‐tubulin (Cell Signaling Technology, 86298), rabbit monoclonal anti‐AKT (Cell Signaling Technology, 4685), rabbit monoclonal anti‐phospho‐AKT (Cell Signaling Technology, 4060), rabbit monoclonal anti‐P70 (Cell Signaling Technology, 34475), rabbit monoclonal anti‐phospho‐P70 (Cell Signaling Technology, 9234), rabbit monoclonal anti‐RPS6 (Cell Signaling Technology, 2217), rabbit monoclonal anti‐phospho‐RPS6 (Cell Signaling Technology, 2211), goat anti‐mouse IgG H&L (YIFEIXUE Biotechnology, YFSA01), goat anti‐rabbit IgG H&L (YIFEIXUE Biotechnology, YFSA02). The other primary antibodies used for western blotting are of the same sources as those used for immunofluorescence staining.

### 2‐Cell Embryo Transfer

Littermates of 6‐week‐old control and ZcKO mice were mated with verified fertile C57BL/6J male mice. On the second day of vaginal plugs, 2‐cells were collected from the mouse fallopian tubes, and the embryos were cleaned and transferred to the fallopian tube of pseudopregnant mice using oral pipette. During the operation, sterile disinfection was paid attention to. After the operation, the mice were returned to the SPF feeding environment for further feeding, and the litter was counted.

### Plasmid Construction and mRNA Synthesis

VPS34, 2× FYVE‐EGFP and mt‐Keima cloned in pBSK vectors for mRNA synthesis were synthesized by Tsingke Biological Technology (Beijing, China). For mRNA synthesis, plasmids were linearized by NotI (TaKaRa, 1623) and purified by Plus DNA Clean/Extraction Kit (GeneMark, DP034P‐300). Then mRNAs were created and tailed using HiScribe T7 ARCA mRNA Kit (NEB, E2065S), purified by a RNA Clean & Concentrator‐25 Kit (Zymo Research, R1017), and stored at −80 °C for future use.

### Microinjection

mRNA for overexpression were injected into oocytes by microinjector (Lecia, Germany). Ten pl of 500 ng µL^−1^ mRNA were injected into oocytes. The injected oocytes were arrested at the GV stage for 2‐10 h in M16 medium containing 2 µM milrinone (Sigma‐Aldrich, 78415‐72‐2) after mRNA injection. Then the oocytes were washed for 5 times and moved to M16 culture medium without milrinone for subsequent experiments.

### Immunoprecipitation

1 × 10^6^ 293T cells or 1600 oocytes were lysed with RIPA lysate containing protease inhibitor on ice for 20 min. A total of 0.5 µg antibodies were added to each protein sample and incubated at 4 °C in a rotating wheel overnight. Then 30 µL protein‐A/G beads (Sigma‐Aldrich, LSKMAGAG) were added into each sample and incubated at 4 °C for 6 h. After centrifugation at 2000 g for 20 s, the supernatants were removed and the beads were washed with cold PBS twice. The beads were then added with protein loading buffer and denatured at 95 °C for 5 min. After a brief quick centrifugation, the collected supernatants were for western blots with specific antibodies.

### GST Pull‐Down Assay

For VPS34‐RAB7 pull‐down assay, the HA‐GST and VPS34‐HA‐GST was expressed as GST‐fusion proteins in E. coli and purified using a GST‐fusion protein purification kit (Beyotime, P2262) following the manufacturer's protocol. The RAB7‐His protein was purified from HEK293 cells 48 h after transfection by immunoprecipitation with anti‐His antibody beads (Beyotime, P2243). ≈100 µg of HA‐GST and VPS34‐HA‐GST fusion protein was immobilized in 50 µL of glutathione agarose and equilibrated before being incubated together at 4 °C for 60 min with gentle rocking motion. ≈100 µg of His‐RAB7 fusion protein was added to the immobilized VPS34‐HA‐GST and HA‐GST after 3 washes with PBS. The fusion proteins were incubated overnight at 4 °C under gentle rotation. The bound proteins were eluted with elution buffer (10 mM glutathione in PBS, pH 8.0) and analyzed by immunoblotting. For active RAB7‐RILP pull‐down assay, the GST‐RILP fusion proteins were prepared and ≈100 µg of GST‐RILP fusion protein was immobilized as described previously.^[^ ^74^ ^]^ The lysate of Vps34^f/f^ and ZcKO oocyte were incubated with immobilized GST‐RILP fusion protein overnight at 4 °C under gentle rotation. The active RAB7 and GST‐RILP were eluted with elution buffer (10 mM glutathione in PBS, pH 8.0) and analyzed by immunoblotting.

### RNA‐seq

Oocytes and embryos were taken out from −80 °C freezer, and thawed on ice, 1 µL of 10 µm oligo‐dT primer, 1 µL of 10 mM dNTP mix, and 0.1 µL of ERCC spike‐in RNA (1:100000 dilution, Life Technologies, 4456740) were added to the lysis buffer. cDNA synthesis and amplification were generated based on Smart‐seq2.^[^ ^62^ ^]^ Sequence libraries were constructed using the KAPA HyperPlus Prep Kit (KK8514) according to the manufacturer's instructions. All libraries were sequenced on an Illumina HiSeq2500. Use fastqc and trim_galore to control read quality. Clean reads were aligned with the mouse genome mm10 using HISAT2 with default parameters. Quantify the read counts using featureCounts. The R package DESeq2 was used for differential expression detection. Genes with p value < 0.05 and a fold change >2 were marked as differentially expressed genes (DEGs). Gene ontology (GO) analysis was performed using DAVID. Major ZGA gene clusters and maternal gene clusters were obtained from DBTMEE database. Fisher's exact test was used for P values of log2(up‐regulated gene/down‐regulated gene) significance between control group and mutant group. The heatmap was produced using the R package pheatmap in. Other plots were prepared using R package ggplot2.

### Transmission Electron Microscopy (TEM)

Oocytes were fixed with 2.5% glutaraldehyde for 2 h and stained with eosin (Bio‐Channel, BC‐DL‐043) for 5 s. Then the oocytes were embedded in 1.5% agarose (BIOFROXX, 1110GR100) for ease of manipulation and the agarose gel cube containing oocytes were fixed overnight in 2.5% glutaraldehyde. The oocytes were then dehydrated through an alcohol gradient and wrapped in epoxypropane resin following standard TEM procedures. Finally, oocytes were observed by transmission electron microscopy (Tecnai G2 Spirit BioTwin, FEI, USA).

### Construction of Vps34 KO Cell Lines

Generation of Vps34 knockout cell lines using CRISPR‐Cas9 gene editing VPS34 knockout on 293T cell lines was carried out by plasmid‐based transfection of Cas9 and gRNA using pST1374 and pGL3 plasmid, respectively. Initially, two sgRNAs for Vps34 were designed using CRISPR direct website (http://crispr.mit.edu) to minimize potential off‐target effects. The sequence of sgRNA is as follows: sgRNA‐1#F, 5ʹ‐ACCGGGTAGCATACCTTAACACAA‐3ʹ; sgRNA‐2#F, 5ʹ ‐ACCGGCTTTGTAGGATGTTCTCAC −3ʹ. 1 µg mL^−1^ puromycine (YEASEN, 60209ES60) and 10 µg mL^−1^ blasticidin (YEASEN, 60218ES10) were selected for drug screening after transfection. The cells were then collected and plated in the DMEM, supplemented with 10% FBS. Single cells were sorted into a 96‐well plate using a limiting dilution method. The forward and reverse primers for this genotyping analysis were, 5′‐ CCTAGAAAGCTGGTGACTTCC‐3′ and 5′‐ TCTGAGCTCCAAACATCAGGT‐3′, respectively. The expanded clonal cells were screened by immunoblotting with VPS34 antibodies.

### Corynoxine B (CB) Rescue Assay

GV oocytes or COCs were isolated from the ovaries of 10‐month‐old mice. for microinjection experiments, mRNA was injected into GV oocytes and then block‐cultured in M2 medium containing 20 µM CB (MedChemExpress, HY‐N0901A) and 2 µM milrinone for 16 h before confocal microscope scanning. For MitoTracker and LysoTracker staining, GV‐stage oocytes were cultured in CB containing 20 µM for 16 h to develop into MII oocytes and for subsequent experiments. For IVF, COCs were cultured in MEMα medium containing 20 µM CB, EGF, 0.3% BSA and 10% FBS for 16 h and incubated in vitro with capacitated sperm for subsequent experiments.

### Fluorescent Intensity Measurement

The strength measurement range is shown in the straight line in the figure. The intensity was measured by ImageJ. The integrated intensity of dot signal was measured with the Plot Profile plugin in ImageJ.

### Statistical Analysis

Each experiment was repeated at least three times. GraphPad Prism software was used to analyze data. Student's *t*‐test one‐way ANOVA, Chi‐square test or Fisher's exact test were used to compare the statistical differences between experimental groups. All data are presented as mean ± SEM. *P* < 0.05 considered to indicate a significant result.

## Conflict of Interest

The authors declare no conflict of interest.

## Author Contributions

W.W.L., K.H.W., and Y.T.L. contributed equally to this work. J.L., F.Y.D., and W.P.X. conceptualized the study, designed experiments, and analyzed data. W.W.L., K.H.W., and J.L. wrote the manuscript. W.W.L., K.H.W., L.W., X.J., Y.X.Q., Y.S., and X.W.D. performed experiments, analyzed data, and prepared figures. W.Y.S. analyzed all the sequencing data. Y.T.L. and L.Z. helped to collect human oocytes and performed all the related experiments. S.M.L. and Q.Y.S. created GFP‐LC3‐ZcKO mice. Y.Q.S. provided Zp3‐Cre transgenic mice and worked on data analysis and paper revision. All authors participated in the preparation of the manuscript.

## Supporting information

Supporting Information

## Data Availability

The data that support the findings of this study are available from the corresponding author upon reasonable request.
