# Supplementary material for: VPS34 Governs Oocyte Developmental Competence by Regulating Mito/Autophagy: A Novel Insight into the Significance of RAB7 Activity and Its Subcellular Location
Source: Adv Sci (Weinh). 2024 Sep 17;11(41):2308823. doi: 10.1002/advs.202308823 (PMC11538714; doi:10.1002/advs.202308823)
Supplement: Supplementary file 1 — Supporting Information [file ADVS-11-2308823-s001.docx]

**Supplementary Figure and Figure Legend**


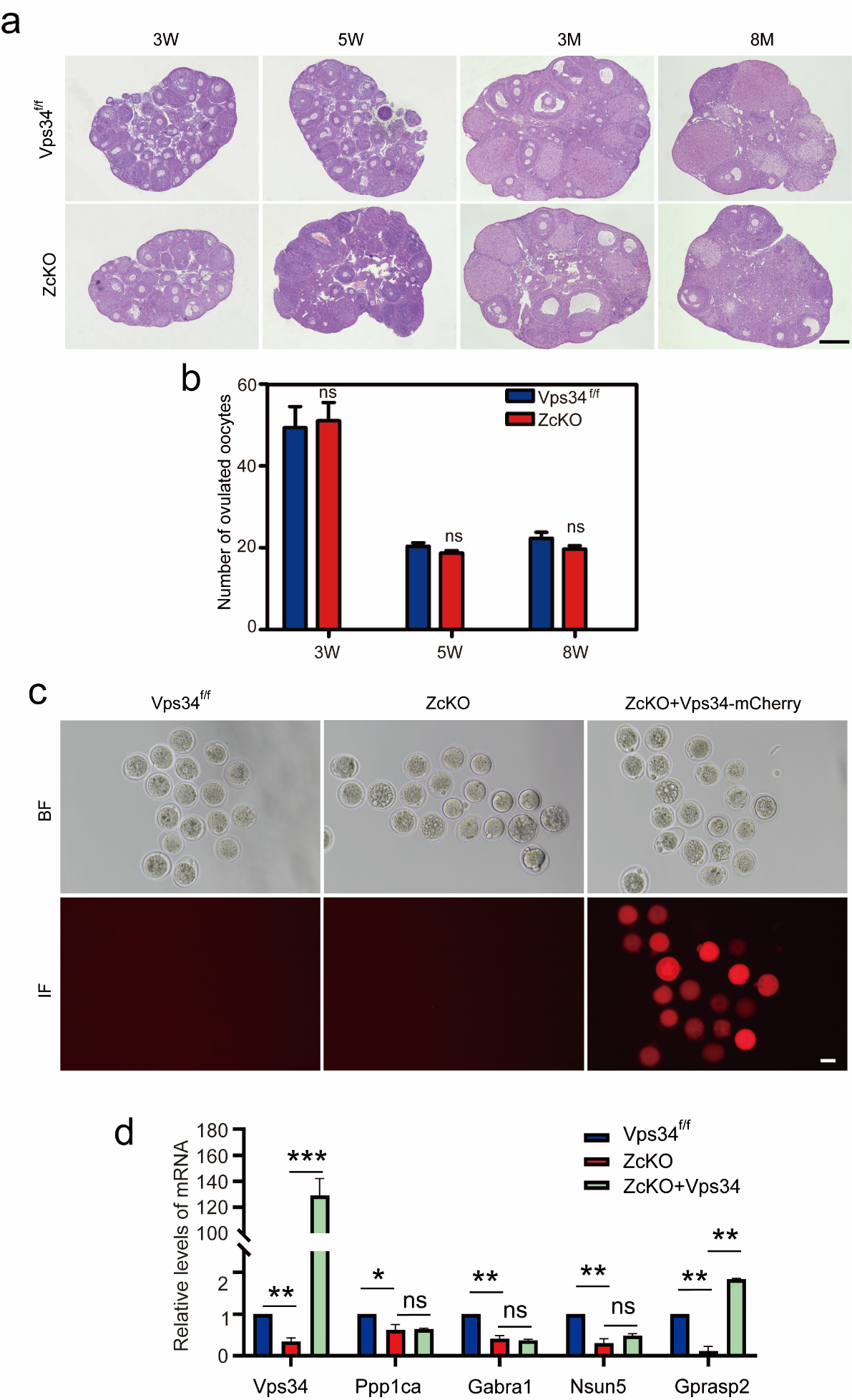
**Supplementary Figure 1.** **Assessment of ovaries and oocytes from control and ZcKO mice.** **a** Ovarian morphology of control and ZcKO mice at 3W, 5W, 3M and 8M of age. Scale bar = 100 μm. **b** Comparison of ovulated numbers between control and ZcKO mice at 3W, 5W and 8W of age. **c** Bright field and fluorescence signals acquired from control zygotes, ZcKO zygotes, and ZcKO zygotes following microinjected with Vps34-mCherry mRNA. Zygotes were collected three hours later after the microinjection. Scale bar = 50 μm. **d** Validation of DEGs associated with ZGA and 2-cell transient activation by RT-PCR in 2-cell embryos of control, ZcKO and ZcKO+Vps34 mRNA groups. Two-cell embryos were collected after twenty-four hours of microinjection. At least 3 independent replicates were performed for each experiment. All graphs are presented as the means ± SEM. *P < 0.05, **P < 0.01, ***P < 0.001. ns = no significance.


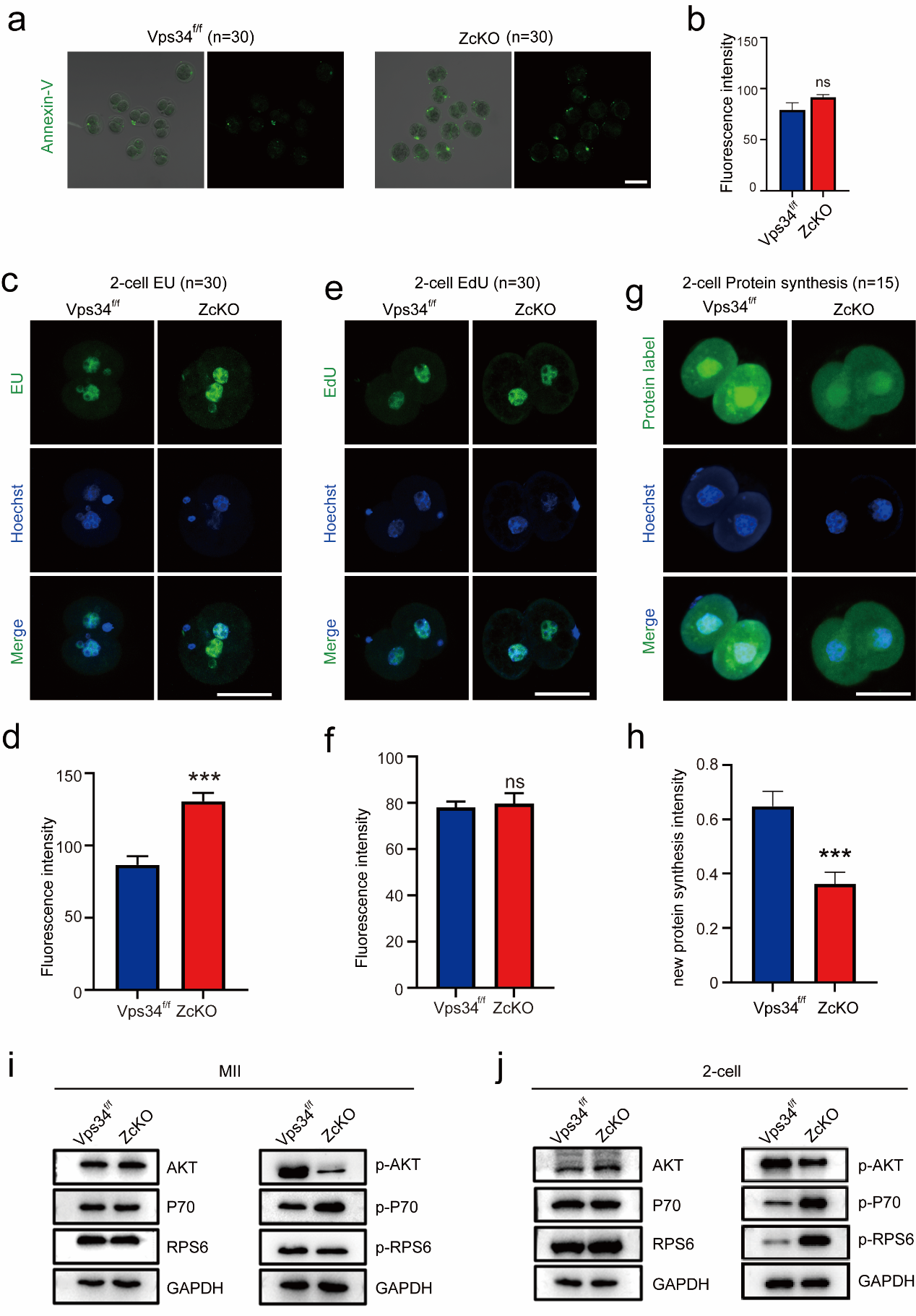
**Supplementary Figure 2. Assessment of 2-cell embryos derived from control and ZcKO oocytes. a** Annexin-V staining on 2-cell embryos derived from control and ZcKO oocytes. The top panels show superimposed images of the GFP fluorescence channel with the bright field. Scale bar = 50 μm. **b** The intensity of Annexin-V fluorescence in 2-cell embryos of the two groups. **d, e** EU staining **(d)** and fluorescence density **(e)** in 2-cell embryos derived from control and ZcKO oocytes. Scale bar = 50 μm. **f, g** EdU staining **(f)** and fluorescence density **(g)** in 2-cell embryos derived from control and ZcKO oocytes. Scale bar = 50 μm. **h, i** HPG labeling of protein synthesis in 2-cell embryos derived from control and ZcKO oocytes **(h)**. Immunofluorescence density of HPG staining **(i)**. Scale bar = 50 µm. **j, k** Western blot of the PI3K/mTOR pathway related proteins in MII oocytes and 2-cell embryos from control and ZcKO mice. GAPDH was used as an internal control. At least 3 independent replicates were performed for each experiment. All graphs are presented as the means ± SEM. *P < 0.05, **P < 0.01, ***P < 0.001. ns = no significance.


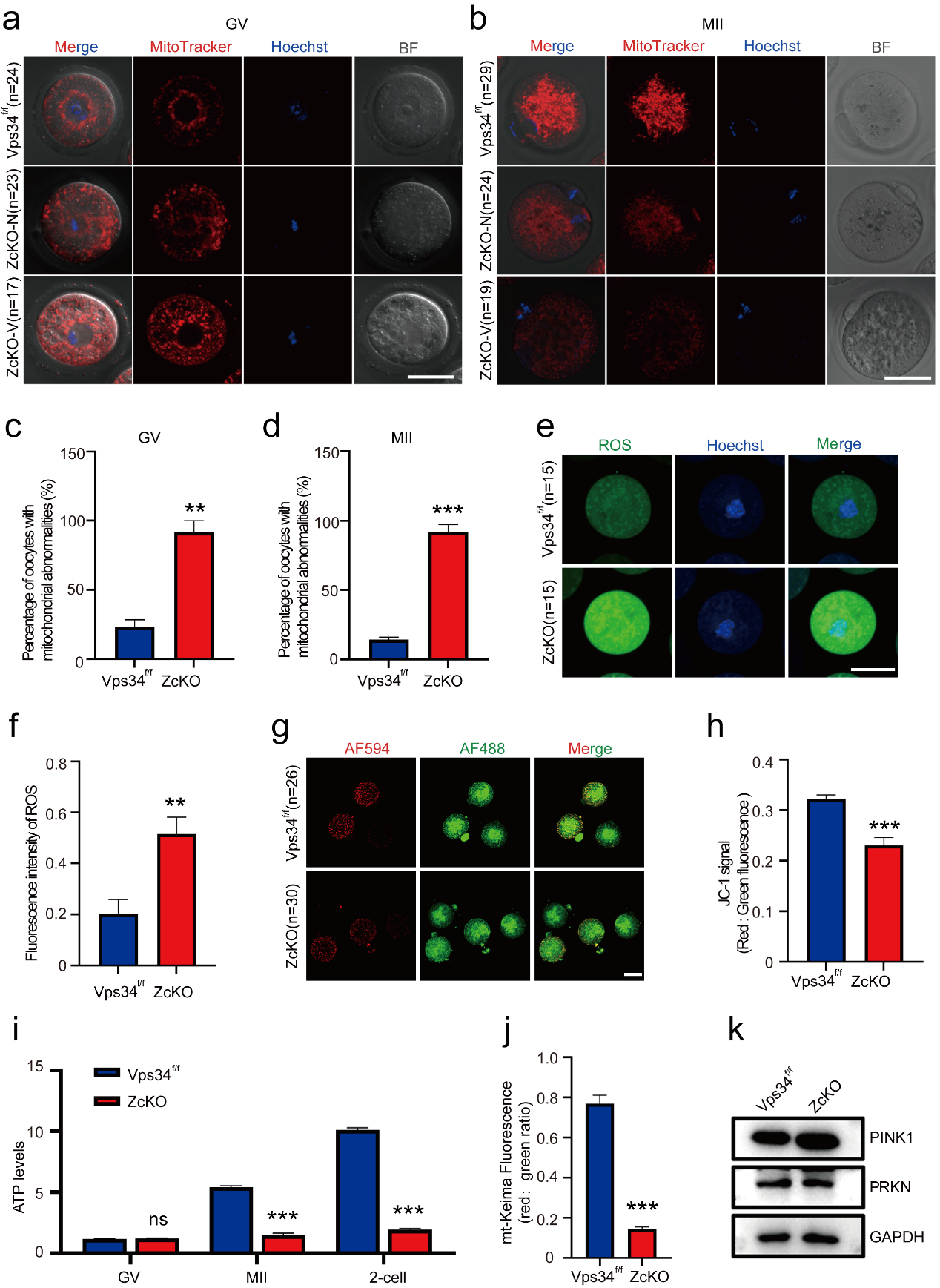
**Supplementary Figure 3. Evaluation of mitochondria functions in oocytes and 2-cell embryos from control and ZcKO mice.** GV and MII oocytes were collected from 5W of control ZcKO mice after superovulation. **a, b** Mitotracker staining of GV **(a)** and MII **(b)** oocytes from control and ZcKO mice. GV and MII oocytes collected from ZcKO mice were classified as non-vacuolated oocytes (ZcKO-N) and vacuolated oocytes (ZcKO-V). Nuclear was stained with Hoechst 33342. BF, bright field; Merge, representing a superposition of fluorescence signals with the bright field. Scale bar = 50 µm. **c, d** Percentage of oocytes with abnormal mitochondria distributions in GV **(c)** and MII **(d)** oocytes from control and ZcKO mice. **e, f** ROS staining **(e)** and fluorescence intensity **(f)** in control and ZcKO GV oocytes. Scale bar = 50 μm. **g, h** Mitochondria membrane potential in control and ZcKO MII oocytes. Mitochondria membrane potential was evaluated by JC-1 staining **(g)** and was measured by the ratio of AF594 (Red) to AF488 (Green) (**h)**. Scale bar = 50 µm. **i** ATP levels in GV, MII oocytes and 2-cell embryos from control and ZcKO mice. n = 30 in each group. **j** Mitophagic activity in control and ZcKO GV oocytes. GV oocytes from control and ZcKO mice were microinjected with mt-Keima mRNAs and hold in 2 μM milrinone for 8-10 h. Images of oocytes were collected at fluorescent emission 550 nm (red) and 440 nm (green), respectively. The ratio of 550/440 fluorescent density was used as an index of mitophagic activity. n = 15 oocytes in each group. **k** Western blot of PINK1 and PRKN in control and ZcKO MII oocytes. GAPDH was used as a loading control. At least 3 independent replicates were performed for each experiment. **P < 0.01, ***P < 0.001. ns = no significance.


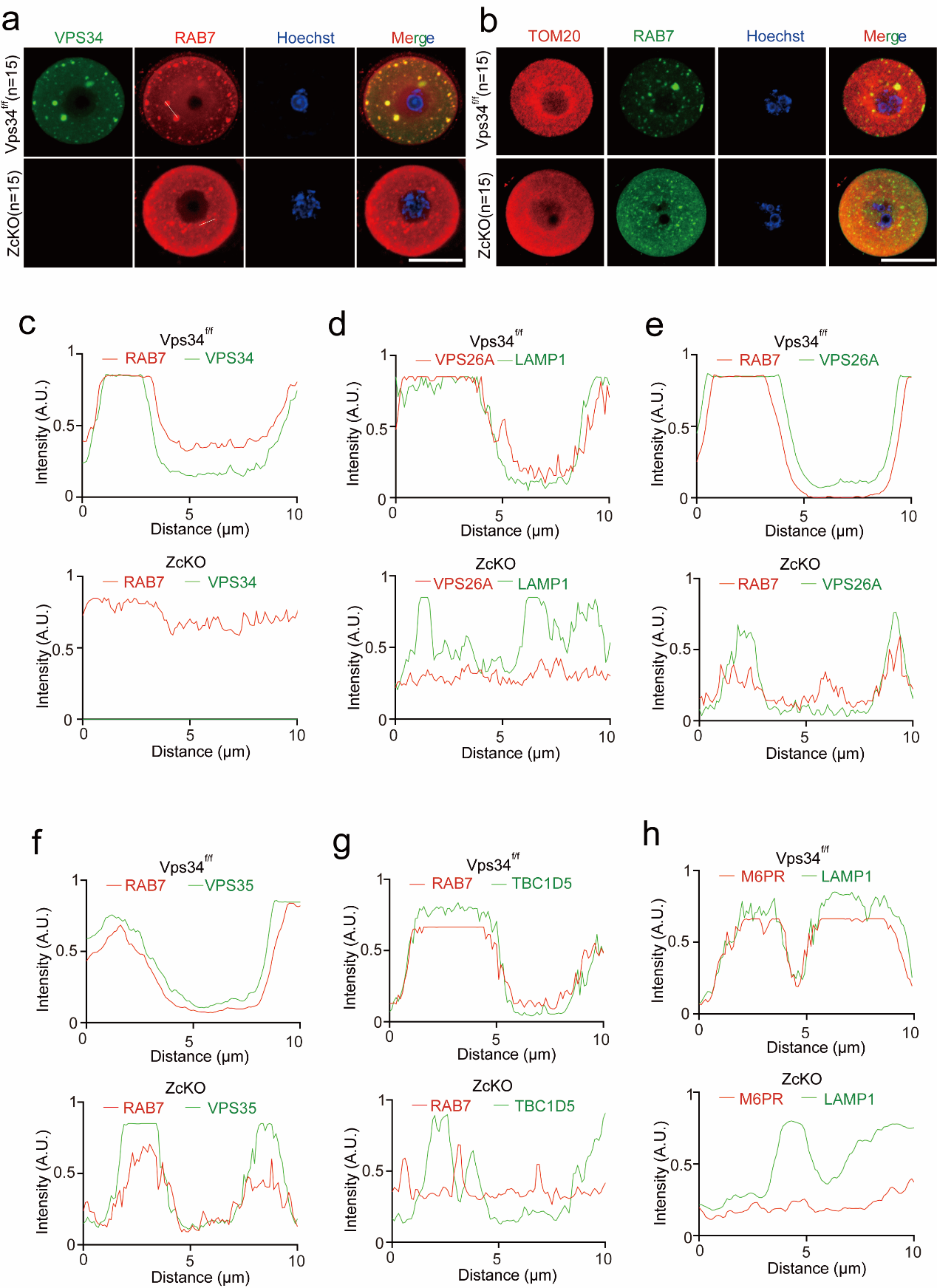
**Supplementary Figure 4. Loss of VPS34 resulted in abnormal localization of RAB7 in oocytes.** **a** Immunofluorescent co-staining of RAB7 (red) with VPS34 (green) in control and ZcKO GV oocytes. Scale bar = 50 µm. **b** Immunofluorescent co-staining of TOM20 (red) with RAB7 (green) in control and ZcKO GV oocytes. Scale bar = 50 µm. **c** Intensity measurement of the lines shown inside the oocytes after co-staining of RAB7 with VPS34 **(a)**. **d-h** Intensity measurement of the lines shown inside the oocytes in Figure 6a-e.


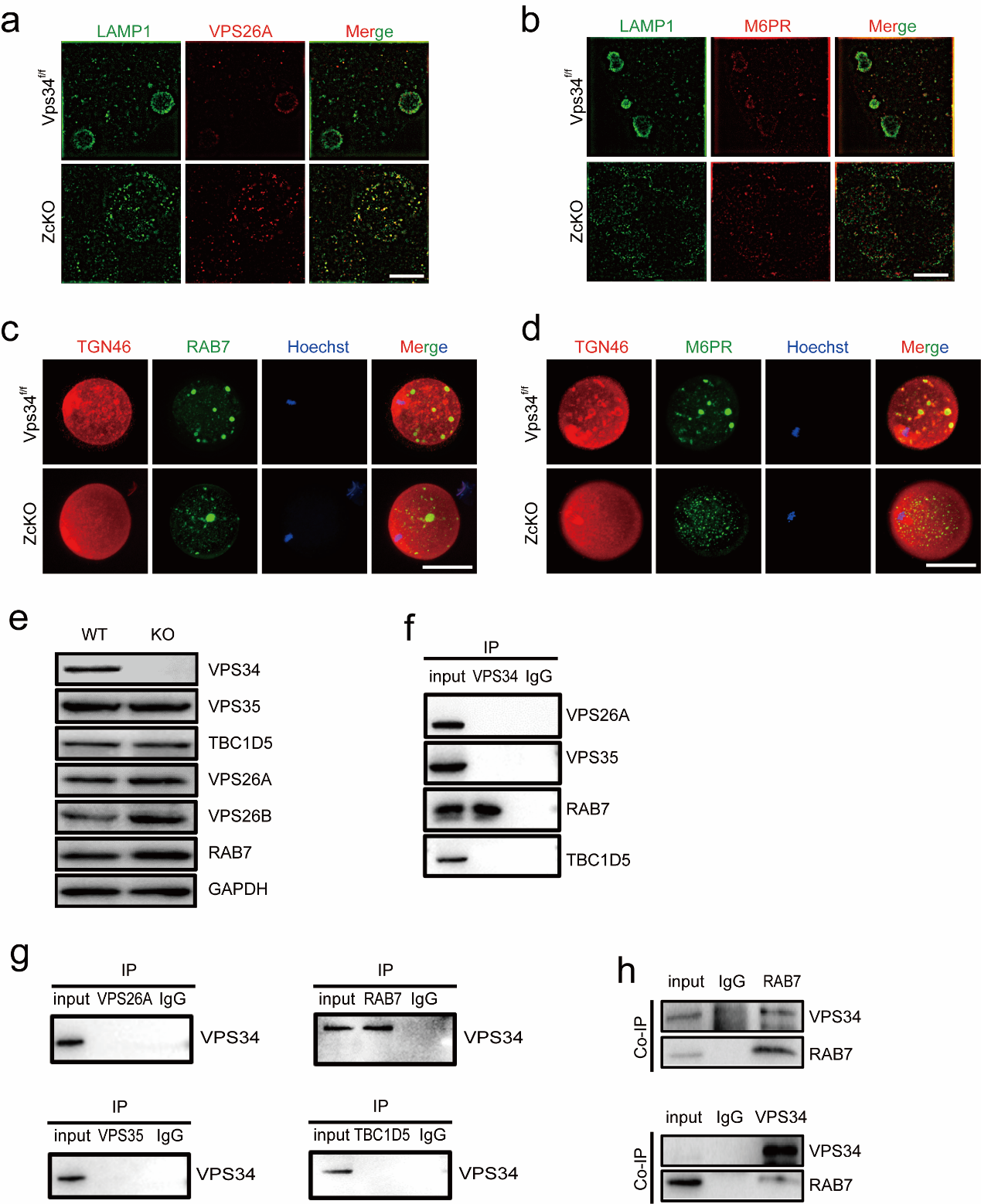


**Supplementary Figure 5.** **Loss of VPS34 resulted in abnormal assembly of retromer complexes in oocytes. a, b** Ultra-high resolution imaging of LAMP1 (green) with VPS26A (red) **(a)** and LAMP1 (green) with M6PR (red) **(b)** co-staining in control and ZcKO MII oocytes. Scale bar=5um. **c, d** Immunofluorescent co-staining of RAB7 (green) with TGN46 (red) **(c)** and M6PR (green) with TGN46 (red) **(d)** in control and ZcKO MII oocytes. Scale bar = 50um. **e** Western blots of retromer components and RAB7 in control and Vps34-knockout (KO) 293T cells. **f** Co-IPs of endogenous VPS34 with components of the retromer complex, VPS26A, VPS35 and RAB7 in 293T cells. **g** Co-IPs of endogenous RAB7 and components of the retromer complex with endogenous VPS34 in 293T cells. Proteins of 293T cells were first immunoprecipitated with antibodies against VPS26A, VPS35, RAB7 and TBC1D5 and their bindings with VPS34 were detected by western blot. **h.** Co-IP assays demonstrated the interaction between RAB7 and VPS34 in oocytes.
